# Carbon xerogels combined with nanotubes as solid-phase extraction sorbent to determine metaflumizone and seven other surface and drinking water micropollutants

**DOI:** 10.1038/s41598-021-93163-2

**Published:** 2021-07-05

**Authors:** Marta O. Barbosa, Rui S. Ribeiro, Ana R. L. Ribeiro, M. Fernando R. Pereira, Adrián M. T. Silva

**Affiliations:** Laboratory of Separation and Reaction Engineering-Laboratory of Catalysis and Materials (LSRE-LCM), Faculdade de Engenharia, Universidade do Porto, Rua Dr. Roberto Frias s/n, 4200-465 Porto, Portugal

**Keywords:** Environmental chemistry, Analytical chemistry, Environmental chemistry, Materials chemistry, Surface chemistry

## Abstract

Carbon xerogels (CXs) were synthesized by polycondensation of resorcinol and formaldehyde, followed by thermal annealing, and subjected to hydrothermal oxidation. Solid-phase extraction (SPE) cartridges were filled with CXs and tested for extraction of metaflumizone and other seven environmental micropollutants (acetamiprid, atrazine, isoproturon, methiocarb, carbamazepine, diclofenac, and perfluorooctanesulfonic acid) before chromatographic analysis. The recoveries obtained with the pristine CX were low for most analytes, except for metaflumizone (69 ± 5%). Moreover, it was concluded that the adsorption/desorption process of the micropollutants performed better on CXs with a less acidic surface (i.e., pristine CX). Thus, cartridges were prepared with pristine CX and multi-walled carbon nanotubes (MWCNTs) in a multi-layer configuration. This reusable cartridge was able to simultaneously extract the eight micropollutants and was used to validate an analytical methodology based on SPE followed by ultra-high performance liquid chromatography-tandem mass spectrometry. A widespread occurrence of 6/8 target compounds was observed in surface water collected in rivers supplying three drinking water treatment plants and in the resulting drinking water at the endpoint of each distribution system. Therefore, the first study employing CXs and MWCNTs as sorbent in multi-layer SPE cartridges is herein reported as a proof of concept for determination of multi-class water micropollutants.

## Introduction

Pesticides, industrial and pharmaceutical compounds, are widely used in diverse activities, and many are indispensable in daily life^1^. However, some of these compounds have been identified as priority substances (PSs, as defined in EU Directive 2013/39)^2^ or contaminants of emerging concern (CECs, as defined in EU Decisions 2015/495, 2018/840, and 2020/1161)^3–5^. The recognized (PSs) or suspected (CECs) impact of these organic micropollutants (OMPs) on the environment and human health have been reported^6–8^, in particular when occurring in surface (SW) and drinking water (DW). OMPs are released in effluents from conventional urban and municipal wastewater treatment plants (WWTPs), which are not designed to eliminate these compounds, and from several other activities (e.g., agriculture, livestock and aquaculture), reaching water bodies and courses in a direct way and/or through surface runoff^9,10^. Therefore, the broad identification and quantification of PSs and CECs in aquatic compartments are essential to compile the data needed to evolve regulatory frameworks in the field of water policy (e.g., transport and fate in the environment and effects on human and ecological health).

Since OMPs are typically found at residual concentrations in the environment (ng L^−1^ to µg L^−1^), it is important to employ an accurate and precise preconcentration step prior to the analysis by using a sensitive and reproducible analytical technique, such as ultra-high performance liquid chromatography-tandem mass spectrometry (UHPLC-MS/MS). Considering the wide resources and great deal of time involved in this task, novel analytical methods would ideally allow: (i) the simultaneous determination of several distinct chemical compounds at trace levels, thus meeting the multi-class purpose; and (ii) shortening the time required for cleanup of the sample matrix and the extraction of analytes, which are generally the most time-consuming analytical steps^1,11^. Solid-phase extraction (SPE) is the most popular sample preparation technique for environmental samples, recognized as a robust and straightforward tool for obtaining sample extracts enriched in target analytes and free of interfering species present in the matrix^12–14^. It is well known that the selection of the most appropriate sorbent is the key step in SPE, since the enrichment efficiency of the target analytes depends on the sorbent characteristics^15,16^. A wide range of sorbents is currently available. Nevertheless, chemically modified silica gel, polymeric sorbents and, more recently, carbon-based materials, are the most commonly used^17–19^.

Carbon xerogels (CXs) can be obtained by sol–gel polycondensation of resorcinol with formaldehyde, followed by conventional drying and thermal annealing under inert atmosphere^20^. CXs present some advantages when compared with other carbon materials, such as the reproducibility of the synthesis procedure and the possibility to control their textural and surface chemistry properties^21^. Furthermore, these materials are characterized by having high porosity and surface area, controllable pore size, and also having the possibility of being shaped as desired^22,23^. These attributes make CXs attractive materials for several applications, including fuel cells^24,25^, catalysis^26–28^ and adsorption for both water treatment^29,30^ or analytical chemistry^31,32^. Regarding water treatment by adsorption, some authors have been studying CXs for removal of specific PSs and CECs: (i) Moral-Rodriguez et al.^33^ studied different CXs for the adsorption of diclofenac; (ii) Álvarez et al.^34^ applied CXs as adsorbents for the removal of caffeine and diclofenac; and (iii) Carabineiro et al.^35^ studied the adsorption capacity of CXs for the antibiotic ciprofloxacin. In the field of analytical chemistry, only two studies were found in the literature for the analysis of pollutants by using CXs in SPE: (i) one to determine the effectiveness of an oxidized CX material as sorbent in SPE to preconcentrate lead from tap water samples^32^; and (ii) another to extract aryloxyphenoxypropionate herbicides from aquatic environmental samples by micro-SPE^31^. However, studies focused on the application of CXs as SPE adsorbent for extraction of other PSs^2^ or CECs listed in the Watch List of the EU Decisions^3–5^ were not found.

In the present work, pristine and hydrothermally modified CXs were synthetized, characterized and applied as SPE adsorbents for the simultaneous extraction of 8 EU multi-class OMPs in water samples, namely 5 pesticides (acetamiprid, atrazine, isoproturon, metaflumizone, and methiocarb), 2 pharmaceuticals (carbamazepine and diclofenac) and 1 industrial compound (perfluorooctanesulfonic acid), followed by analysis with UHPLC-MS/MS. SPE cartridges packed with multi-walled carbon nanotubes (MWCNTs) performed well for 7 out of these 8 target compounds in our previous study^36^, but failed to recover metaflumizone, which is included in the very recent Watch List of EU Decision 2020/1161^5^. Therefore, upon broadening the range of carbon materials explored, the goal of the present study was to find a sorbent with a good performance for the recovery of metaflumizone, aiming at the development of a SPE carbon-based cartridge in multi-layer configuration performing well for all the 8 OMPs.

Additionally, an analytical method based on SPE-UHPLC–MS/MS was validated with the optimized multi-layer carbon cartridge and used to assess the occurrence of the 8 multi-class contaminants in SW samples strategically collected at different locations (i.e., near the intake of 3 drinking water treatment plants—DWTPs—in northern Portugal) and in the DW yielded by those plants. To the best of our knowledge, this is the first time a (non-polymeric) multi-layer carbon cartridge was prepared for the extraction and preconcentration of OMPs. Therefore, the novelty of the present work is based (i) on the development of a multi-layer carbon-based SPE cartridge using both CXs and MWCNTs as sorbents (i.e., the first non-polymeric multi-layer carbon cartridge to determine OMPs); and also (ii) on the application of this innovative cartridge to validate a SPE-UHPLC-MS/MS method, able to simultaneously determine metaflumizone and other relevant PSs and CECs defined in recently launched EU legislation.

## Experimental section

### Chemicals and materials

Formaldehyde solution (37 wt% in water, stabilized with 15 wt% of methanol), resorcinol (99 wt%), and sodium thiosulfate were purchased from Sigma-Aldrich (Steinhein, Germany). Commercial MWCNTs Nanocyl 3100 (NC3100, powder), with an average diameter of 9.5 nm, average length of 1.5 µm and > 95% purity were purchased from Nanocyl SA (Sambreville, Belgium). The reference standards (acetamiprid, atrazine, carbamazepine, diclofenac sodium, isoproturon, metaflumizone, methiocarb and perfluorooctanesulfonic acid—PFOS; > 98% purity) and deuterated substances used as surrogate standards (acetamiprid–d3, atrazine–d5, diclofenac-d4, and methiocarb-d3) were acquired from Sigma-Aldrich (Steinhein, Germany). The properties of these analytes (i.e., class, structure, relative molecular mass (M_r_), p*K*a, and log *K*_OW_ values and solubility in water) can be found in the Table S1 of the Supplementary Material. Hydrochloric acid, ethanol (HPLC grade), and methanol (MS grade) were purchased from VWR International (Fontenay-sous-Bois, France). The stock solution and the working solutions were prepared according our previous work^1,36^. Briefly, the stock solution was prepared in ethanol with 1000 mg L^−1^ of each reference or internal standard and the working solutions comprising all the target compounds or the internal standards (2.5 mg L^−1^ and 5.0 mg L^−1^, respectively) were prepared by dilution of the individual stocks in ethanol. Sodium chloride was supplied by JMGS (Odivelas, Portugal). Sodium hydroxide, nitric acid and sulfuric acid were acquired from Merck (Darmstadt, Germany). Empty SPE cartridges (6 mL) with two frits (20 µm) (Bond Elut) were purchased from VWR International (Fontenay-sous-Bois, France). Ultrapure water was provided by a Milli-Q water system, whereas a pHenomenal pH 1100L pH meter (VWR, Germany) was used for all the pH measurements.

### Preparation and modification of carbon materials

Two types of carbon materials were used in this work, namely in-house prepared CXs and commercial MWCNTs. More details (i.e., textural and surface chemistry characterization) on the MWCNTs can be found in our previous publication^36^. The pristine CX material was prepared by polycondensation of resorcinol with formaldehyde using a molar ratio of 1:2, adapting the procedures described elsewhere^20,22^. The sol–gel step was carried out at pH 6.0, sodium hydroxide solutions being added dropwise under continuous stirring until achieving the desired pH. This step is crucial since the accurate control of pH was determinant for the development of the mesoporous character of CXs^22^.

The original CX material was modified by surface functionalization with HNO_3_, following the hydrothermal procedure described elsewhere^37^. Specifically, the hydrothermal oxidation was conducted in a Teflon-lined stainless-steel autoclave (125 mL, Mod. 4748, Parr Instruments, USA). The HNO_3_ solution (75 mL, at five different concentrations varying from 0.01 to 0.30 mol L^−1^) was transferred to the PTFE vessel and 0.2 g of CX was loaded. The PTFE vessel was placed inside the stainless-steel autoclave, which was sealed and kept in an oven at 200 °C during 2 h. Subsequently, the autoclave was allowed to cool down until room temperature. The recovered CX sample was washed with distilled water until a neutral pH of the rinsing water was reached and then dried overnight at 120 °C. Moreover, a blank hydrothermal treatment was performed with distilled water instead of the HNO_3_ solution. The six resulting materials were denoted as CX followed by a subscript number corresponding to the concentration of HNO_3_ employed in mol L^−1^, namely: CX_0_, CX_0.01_, CX_0.05_, CX_0.10_, CX_0.20_, and CX_0.30_.

### Characterization of carbon materials

The textural properties were determined from N_2_ adsorption–desorption isotherms at – 196 °C, as described in our previous work^22^ and included the following parameters: specific surface area (*S*_BET_), non-microporous specific surface area (*S*_meso_) and total pore volume (*V*_total_). Thermogravimetric analysis (TGA) was performed in a Netzsch STA 490 PC/4/H Luxx thermal analyser, in which the CX samples were heated from 50 to 900 °C at 10 °C min^−1^, under an inert (N_2_) gas flow. Temperature programmed desorption (TPD) was performed in a fully automated AMI-300 Catalyst Characterization Instrument (Altamira Instruments) with a quadrupole mass spectrometer (Dymaxion, Ametek), as described elsewhere^37^. The pH at point of zero charge (pH_PZC_) was determined by pH drift tests^37^.

### Adsorption and desorption experiments

Batch adsorption and desorption experiments were carried out individually with the oxidized and pristine CXs and MWCNTs, and the 8 target OMPs. Batch adsorption studies were conducted in volumetric flasks containing 125 mL of filtered surface water at natural pH (around 7) spiked with a known concentration of the 8 target OMPs. 12.5 mg of each carbon material (i.e., original CX and MWCNT samples and 0.3 mol L^−1^ of HNO_3_ hydrothermally treated CX and MWCNT samples) was transferred into the respective flask at the starting time and the flasks were placed in a platform shaker at 200 rpm and 24 °C. Small volumes of samples (without carbon material in suspension) were periodically taken from the flasks (at times: 0, 5, 30, 60, 120, 180 and 300 min), filtered through 1.2-μm glass-fiber filters (47 mm GF/C, Whatman, Maidstone, United Kingdom) and placed in a glass vial for analysis. Immediately after these batch adsorption tests, the content of each volumetric flask (containing adsorbent and adsorbates) was dumped in a centrifuge tube and the adsorbent was separated from the aqueous solution by centrifuging at 3200 rpm (5 min). To evaluate the desorption process, the target analytes were extracted by exposing the recovered carbon materials to the eluting solvent (ethanol) under continuous stirring during 10 min; then, 1 mL sample was taken, filtered through 1.2-μm glass-fiber filters (47 mm GF/C, Whatman, Maidstone, United Kingdom) and transferred to a vial for subsequent analysis. Each experiment was conducted in triplicate. Blank tests (i.e., not including the carbon material samples) were also carried out. During the adsorption and desorption experiments, no significant changes in temperature were observed. All the samples were analysed by UHPLC-MS/MS.

### SPE procedure

Handmade cartridges with single (i.e., containing a CX sample only) and multi-layer configuration (i.e., with both CX and MWCNT) were packed by using a device specially created for that purpose^36^. This process comprises several successive phases, namely: (i) a polyethylene frit (20 µm) was positioned on the bottom of an empty SPE cartridge (6 mL); (ii) a selected amount of carbon material was then introduced in the cartridge; (iii) the carbon sample was protected with another frit; and (iv) a slight compression was applied until a specific bed height was achieved. In the case of multi-layer carbon cartridges, the steps (ii) and (iii) were repeated, to introduce the second layer of sorbent material.

The SPE procedure applied is described in our previous works^1,36^. Briefly, the conditioning solvents ethanol or methanol and ultrapure water were successively passed through the SPE cartridge at a flow rate of 1 mL min^−1^. Sample loading of blank (i.e., without spiking of the analytes of interest) or spiked (200 ng L^−1^ of each target analyte) water samples was performed at a constant flow rate of 10 mL min^−1^ and 4 mL of ultrapure water were used in the washing step. After drying under vacuum (45 min), the analytes were eluted with ethanol or methanol and the extracts were evaporated to dryness in a Centrivap Concentrator device (LABCONCO Corporation, Kansas City, MO, USA). After reconstitution of these dried extracts in ethanol (250 µL), as described elsewhere^36^, the samples were analysed by UHPLC-MS/MS.

The design of experiments (DoE) and data analysis employed for the optimization of SPE with CX-cartridges were accomplished using Minitab 19 (Minitab Statistical Software, Pennsylvania, USA) and the statistical support was achieved by analysis of variance (ANOVA) tests (significant at the 5% level) (Table S2). All experiments carried out during the SPE optimization study were performed in triplicate, the obtained relative standard deviations (RSD) being represented as error bars in the corresponding Figures.

### UHPLC-MS/MS methodology

The water sample analysis was carried out following a methodology published elsewhere^36^, using an UHPLC-MS/MS apparatus (Shimadzu Corporation, Tokyo, Japan) consisting of a Nexera UHPLC equipment with a Ultra-Fast Mass Spectrometry series LCMS-8040 triple quadrupole mass spectrometer. The chromatographic separation conditions and the MS parameters can be consulted in Text S1 of Supplementary Material. The electrospray ionization source operated under both positive and negative ionization modes. Selected reaction monitoring (SRM) was applied to quantify and confirm the identity of each target compound, by using SRM1 for quantification and the ratio between SRM1 and SRM2 for confirmation, along with the retention time of the analyte (Table S3 of the Supplementary Material).

### Quality assurance and control of the analytical method

The validation of the SPE-UHPLC-MS/MS methodology was implemented in accordance with the international guidelines^38^ and the previous works developed by our group^1,39^. The evaluation of the selectivity, linearity and range, instrument and method limits of detection and quantification, accuracy, precision, recovery, matrix effect, and process efficiency, were performed. These results are detailed in the Supplementary Material (Tables S4 and S5).

The matrix effect was calculated as the ratio of: (*A*) the peak areas obtained for blank extracts spiked after SPE, subtracting those of the non-spiked blanks and (*B*) the peak areas of the standards solution with a similar concentration as the post-spiked extracts—Eq. (1)^1^.

1 $$\text{Matrix}\;\text{effect}\;(\%)=100 \times \left(\text{A}/\text{B}\right)$$

The recovery efficiency, i.e., the parameter that supports the selection of the optimum conditions for the SPE procedure, was calculated according to Eq. (2).

2 $$\text{Recovery}\;\text{efficiency}\;(\%)=100 \times \left(\text{C}/\text{A}\right)$$

The recovery (%) was calculated as the ratio of: (*C*) the peak areas obtained for the extracts of a a blank sample spiked before SPE and (*A*) the peak areas of the post-spiked extracted blank sample, subtracting to both areas those of the non-spiked blanks. This approach for evaluating the recovery efficiency allows obtaining only the recovery provided by the adsorbent material, as the matrix effect is considered the same in *C* and *A*^36^. Figure S1a,b show the Total Ion Current (TIC) chromatograms of the 8 target OMPs (200 ng L^−1^) after SPE of a spiked sample and after post-spiking a blank extract using multi-layer carbon-based cartridges (bottom: 25 mg of CXs; top: 25 mg of MWCNTs), in comparison to an ethanolic standard solution (Figure S1c).

The overall performance of the analytical method was assessed by the process efficiency parameter, obtained by Eq. (3):

3 $$\text{Process}\; \text{efficiency}\; (\%)=100 \times \left(\left(\text{A}/\text{B}\right) \times \left(\text{C}/\text{A}\right)\right)=100 \times \left(\text{C}/\text{B}\right)$$

Process efficiency considers the efficiency of sample preparation (i.e., recovery) and analyte ionization (i.e., matrix effect). The process efficiency values are shown in Table S5 of the Supplementary Material.

### Water samples collection and preparation

SW samples specifically used in the optimization of the SPE method were collected from the Cavalum River located in Penafiel (40 km from Porto, Portugal), a tributary of the Sousa River. SW and DW samples used in the monitoring campaign were collected from 3 regions within the northwest of Portugal (served by 3 different DWTPs), in the period of August to October 2020. Specifically, SW was collected near the intake of each DWTP, while DW was collected at an endpoint of the distribution system served by the corresponding plant and subjected to chlorination step in the distribution network. All the SW samples (up to 3 L) were collected away from the shoreline (at the center of the river basin) to avoid stirring up sediments and provide the most representative sample of the entire water body, by dipping a bottle sampler (pre-washed in the laboratory and pre-cleaned with the water from that site) between 10 to 25 cm below the surface. Regarding DW, each tap was open to allow the water to purge for 1 min before sample collection into a bottle sampler^40^. Then, the water samples were stored at 4 °C in amber glass bottles with 1 L of capacity until extraction (24 h). The samples were filtered through 1.2-μm glass-fiber filters (47 mm GF/C, Whatman, Maidstone, United Kingdom) and, when needed, the pH was adjusted before the SPE step. Sodium thiosulfate was added to each DW sample (30 mg L^−1^) to reduce any residual chlorine that might be added as a disinfectant in the DW supply^1^.

## Results and discussion

### Optimization of SPE procedure with pristine CX-cartridges

There are several factors that can affect the efficiency of SPE, namely the mass of sorbent material (mg), sample volume (mL), sample pH, type of solvent and solvent volume (mL). Therefore, a multivariate approach is recommended for this type of optimization involving a large number of parameters. However, some of the parameters mentioned above may not significantly affect the extraction efficiency, so its use in the analysis may be avoided. Thus, a screening study, preceding the optimization step, would be helpful to evaluate the significant factors involved in the analytical sample preparation method. With that goal in mind, a definitive screening design (DSD) was employed for the optimization of SPE with CX-cartridges. The DSD, which involved 14 randomized experiments with 5 factors (mass of sorbent material (mg), sample volume (mL), sample pH, type of solvent and solvent volume (mL)) and 3 levels (− 1, 0 and 1), was applied in a first stage aiming for the determination of the main parameters affecting the recovery efficiency of the 8 target OMPs. Afterwards, a multiple parameter optimization with Box-Benkhen Design (BBD) was performed considering only the significant variables to find the best operating conditions with a minimum number of assays. A three factor BBD with 15 unique sets (random order) and a triplicate on the center point was used. The designated coded values for the variables, − 1, 0 and 1, were used to represent low, middle, and high levels, respectively, and the coded and actual levels of the variables are listed in Table S2, as well as the quadratic polynomial model (Equation S1) used in this method to analyze the results from the BBD.

The DSD results (Table S6) obtained for the 8 target compounds were analysed using Pareto charts (Figure S2), which are very useful as they allow ordering the input factors by significance^41^. In general, the amount of CX packed in the cartridge (factor A), the volume of sample (factor B) and the sample pH (factor C) were the most determining factors for the recovery of the target compounds. In the specific case of acetamiprid (Figure S2a), no factor was significant due to the low recoveries obtained. Overall, the results of this first screening revealed that 3 significant factors (A, B and C) should be considered in the optimization step and the 2 remaining factors should be fixed (i.e., factor D was selected as ethanol and factor E was set to 8 mL of solvent).

BBD was then used (Table S7) to determine the optimum operating conditions upon visual analysis of the corresponding response surfaces and contour plots, and ANOVA (an example of these results are shown in Figure S3 and Table S8 for metaflumizone). To obtain the surfaces, 1 variable is always fixed. The results revealed that the regions of maximum responses for all target compounds correspond to a mass around 50 mg of CX sample (factor A), 1000 mL of water sample (factor B) and natural sample pH (factor C). These values were thus fixed and used in subsequent SPE tests.

### Textural and surface chemistry characterization of CXs

**Figure 1:**
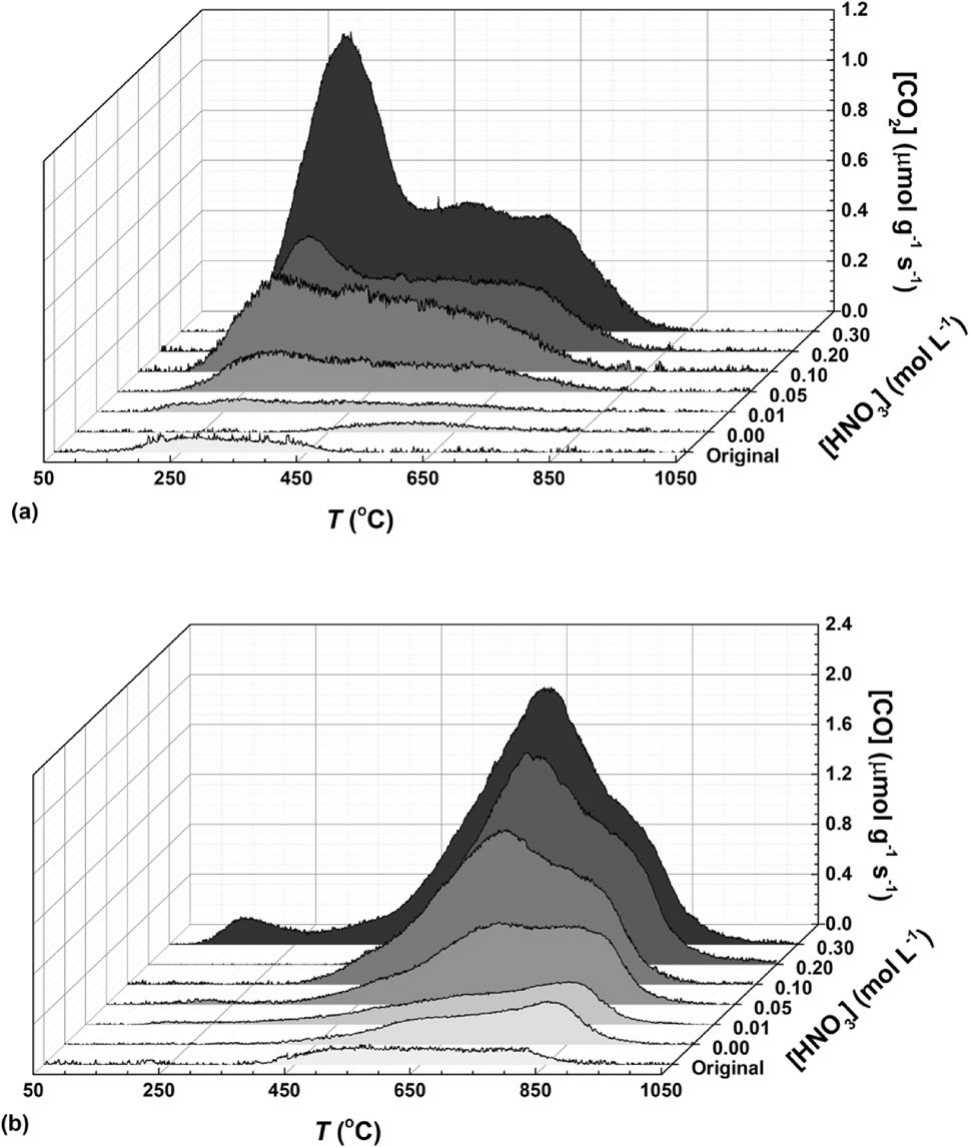
TPD spectra of CXs subjected to hydrothermal treatment with different HNO_3_ concentrations: (**a**) CO_2_ and (**b**) CO evolution with temperature (this figure was produced using OriginPro 9.0.0 software, OriginLab Corporation; http://www.OriginLab.com).

Figure 1a,b show the respective CO_2_ and CO TPD spectra obtained with the hydrothermally treated CXs (HNO_3_ concentration in the range 0.01–0.30 mol L^−1^). The TPD profiles of the pristine CX material (original) and CXs after hydrothermal treatment with water (i.e., [HNO_3_] = 0 mol L^−1^) are also shown for comparison purposes. The total amount of surface groups released as CO_2_ and CO, and the corresponding oxygen content are given in Table 1.

**Table 1:** Properties of the pristine CX material and CXs subjected to hydrothermal treatment with different HNO_3_ concentrations: amounts of CO_2_ and CO released by TPD, [CO/CO_2_] ratio, percentage of oxygen obtained from the analysis of the TPD spectra (assuming that all the surface oxygen is released as CO and/or CO_2_), amount of volatiles (determined by TGA), pH at the point of zero charge (pH_PZC_), specific surface area (*S*_BET_), non-microporous specific surface area (*S*_meso_) and total pore volume (*V*_total_).

[HNO_3_] (mol L^−1^)	Parameters								
	[CO_2_] (μmol g^−1^)	[CO] (μmol g^−1^)	O (wt%)	[CO]/[CO_2_]	Volatiles (wt%)	pH_PZC_	*S*_BET_ (m^2^ g^−1^)	*S*_meso_ (m^2^ g^−1^)	*V*_total_ (cm^3^ g^−1^)
(pristine CX)	174	626	1.6	–	2.32	7.4	699	256	1.267
0 (Blank)	112	1173	2.2	–	3.27	6.9	672	249	1.205
0.01	247	1326	2.9	5.4	4.80	6.6	637	246	1.225
0.05	770	2718	6.8	3.5	9.15	3.9	629	242	1.199
0.10	1752	4359	12.6	2.5	14.0	< 2	716	249	1.247
0.20	1965	5357	14.9	2.7	20.3	< 2	810	268	1.356
0.30	3920	7097	23.9	1.8	25.2	< 2	797	287	1.337

Pristine CXs and the CX treated with water ([HNO_3_] = 0) have low amounts of oxygen surface groups (ca. 2 wt%), as shown in Table 1. The increase in the concentration of HNO_3_ (i.e., the oxidizing agent) leads to a rise in the amounts of CO_2_ and CO released by TPD (Fig. 1a,b, and Table 1) and, as a consequence, the oxygen contents also increase (Table 1). This trend proves that CXs are appropriate carbon materials for the incorporation of oxygenated functional groups through hydrothermal oxidation with HNO_3_ under mild conditions. Moreover, the [CO]/[CO_2_] ratio is above 1 for all the CX samples (Table 1). Thus, the surface groups released as CO are dominant over those released as CO_2_.

**Figure 2:**
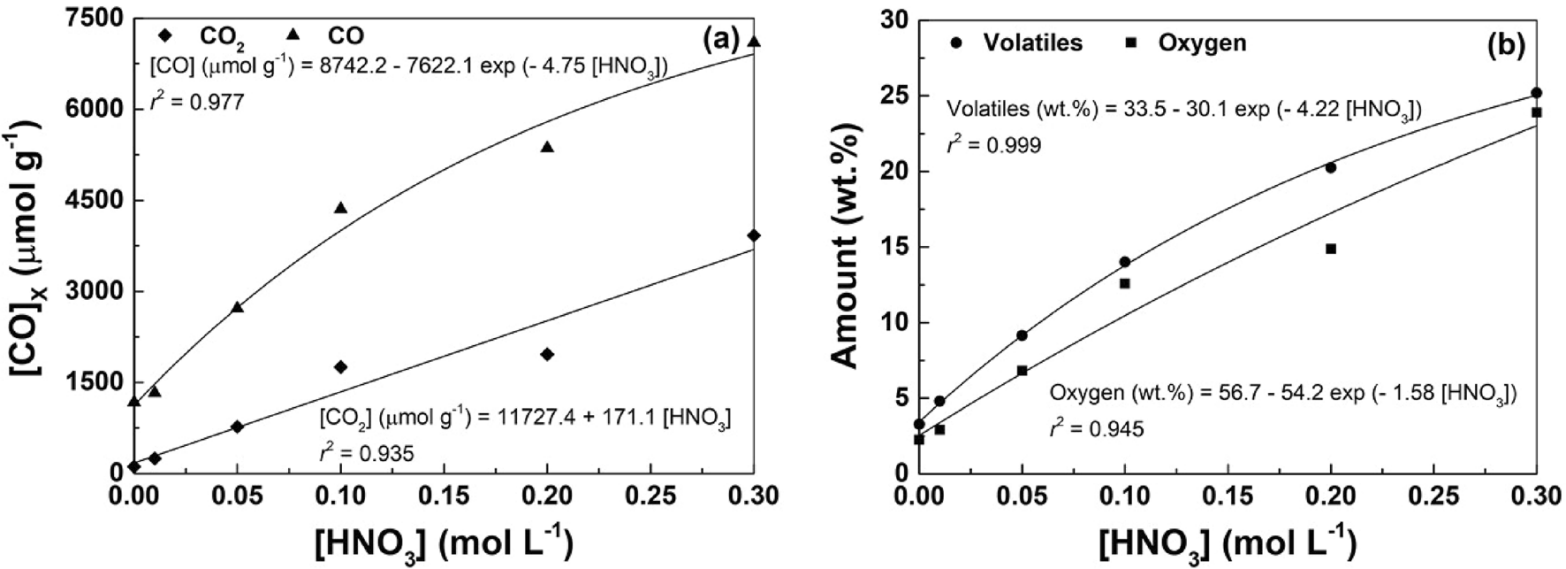
(**a**) Amounts of CO_2_ and CO released by TPD, and (**b**) contents of volatiles and oxygen as a function of the concentration of HNO_3_ employed in the hydrothermal treatment of CXs. Points represent experimental data, while lines represent non-linear fittings.

The amount of oxygenated groups released as CO_2_ and CO and the contents of oxygen and volatiles (determined by TGA under inert atmosphere; Table 1) were correlated with the concentration of HNO_3_ used in the hydrothermal treatment of CX. As observed, single exponential functions can correlate the evolution of all parameters under study as a function of the HNO_3_ concentration (Fig. 2a,b). The correlations found are helpful to tune the amount of oxygenated surface groups introduced, i.e., by fixing the appropriate concentration of HNO_3_ in the hydrothermal treatment. These results are in agreement with our previous studies on hydrothermally treated CXs^22^, MWCNTs^42^ and single-walled CNTs^43^.

**Figure 3:**
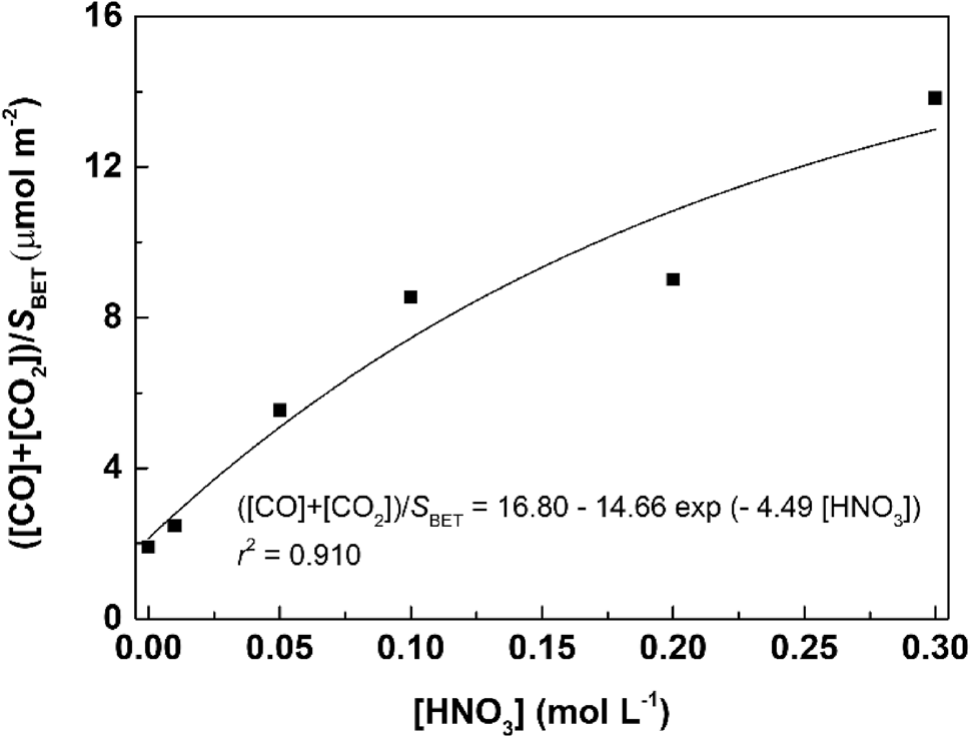
([CO_2_] + [CO])/*S*_BET_ as a function of HNO_3_ concentration.

When the total amounts of CO_2_ and CO are normalized by the *S*_BET_ (i.e., ([CO_2_] + [CO])/*S*_BET_), and represented as a function of HNO_3_ concentration (Fig. 3), a good correlation is also obtained.

The effect of the hydrothermal treatment on the overall surface charge of the resulting samples was studied by pH_PZC_ measurements (Table 1). The results revealed the markedly acidic nature of the CX samples treated with [HNO_3_] ≥ 0.10 mol L^−1^, as inferred from the values of pH_PZC_ < 2.

Regarding the textural properties of the CX materials, the effect of the hydrothermal treatment was evaluated through N_2_ adsorption–desorption isotherms. In general, the surface area (both *S*_BET_ and *S*_meso_) and pore volume (*V*_total_) of CXs increase as the concentration of oxidizing agent used in the hydrothermal treatment increases (Table 1). This slight change of the porous structure of the carbon material under study can be ascribed to the creation of new pores, or opening of previously inaccessible pores, due to some structural defects resulting from the functionalization methodology^22,36^. The mesoporous character of all these materials can be confirmed by the high rise of adsorption of N_2_ at high relative pressures (Figure S4).

### Application of pristine and functionalized CXs for extraction of EU multi-class OMPs

The pristine and hydrothermally treated CXs were tested as adsorbents for extraction of the 8 target EU-relevant OMPs. The effect of surface chemistry and textural properties of the CX samples were evaluated in the adsorption/desorption process of these compounds by comparing the recovery efficiencies of the target compounds calculated by using Eq. (2) (“Quality assurance and control of the analytical method” section). The results showed that low recoveries are obtained with the original CX material for most compounds, except for metaflumizone (Figure S5). Moreover, it was found that, in general, the HNO_3_ hydrothermal treatment has a negative effect on the recovery of the target OMPs (Figure S5). Other solvents (ethanolic solutions of 5% NH_4_OH or 2% of CH_2_O_2_) and a second elution step were tested (Figure S6) in an attempt to improve the recoveries obtained with the original CX material. However, no improvement was observed, and the subsequent tests were thus performed with the previous conditions (8 mL of an eco-friendly solvent, ethanol).

The PSs and CECs under study belong to different classes and possess different physicochemical properties (Table S1). Thus, the mechanisms controlling the adsorption/desorption of these compounds on CXs can be distinct. The main interactions comprise: (i) electrostatic interactions due to the charged carbon material surface; (ii) π–π interactions (between bulk π systems existing on the surface of the adsorbent material and the organic molecules with their benzene rings) or C=C double bonds; and (iii) hydrogen bonds with functional groups on the surface of the CX material^44,45^. It is important to highlight that each of the interactions described above can be affected by the different constituents of environmental matrices. Therefore, performing a mechanistic study on the extraction of multi-class OMPs from real water matrices is a challenge. Nevertheless, the results obtained when employing the pristine and functionalized CXs as SPE sorbents suggest that a less acidic surface (i.e., with a lower amount of surface oxygen-containing groups) favors the adsorption/desorption process of the target OMPs (Figure S5). This trend is clearly identified in the specific case of the pesticide metaflumizone (Figure S7) when the recoveries obtained for this CEC are plotted as a function of ([CO_2_] + [CO])/*S*_BET_. These results suggest that a higher amount of oxygen-containing surface groups may promote a stronger adsorption of the compound on the carbon material, thus hampering the desorption process. In order to explore this hypothesis, batch adsorption and desorption experiments were carried out individually with the oxidized and pristine CXs, and the 8 target OMPs (Table S9). Accordingly, an adsorption over 90% was obtained for all compounds when using both pristine and oxidized CX (0.3 mol L^−1^ of HNO_3_). On the contrary, the highest desorption efficiency was obtained with the original CX sample, in particular for metaflumizone and PFOS, which were the target analytes that yielded the highest SPE recoveries (Table S9). Adsorption and desorption experiments were also performed with MWCNT, a similar trend being observed (Table S9). These results suggest that the desorption step is the bottleneck stage of the SPE process when using both CXs and MWCNTs. Therefore, the desorption step may be considered the main challenge for the application of SPE carbon-based cartridges for extraction of different organic compounds. Comparing the results obtained in the present study with those obtained with MWCNTs in our previous work^36^, it is possible to conclude that the MWCNTs are a more effective option as SPE sorbent for 7 out of the 8 target OMPs. Nevertheless, the CX-cartridges employed in the present study enable a better performance for the extraction of metaflumizone (recovery > 60%; against 2% obtained in the previous study with MWCNTs)^36^. These results can be explained by the lower desorption efficiency obtained with pristine MWCNT in comparison to that obtained with pristine CX (i.e., 0% vs 54%; Table S9). These insigths prompted the study reported in the following Section, i.e., the application of a multi-layer carbon-based cartridge to extract the 8 target OMPs from water samples.

### Multi-layer carbon-based cartridges for determination of EU multi-class OMPs: a proof of concept

In view of the results presented above, we decided to develop a multi-layer carbon-based SPE cartridge with high selectivity/specificity for adsorption/desorption of the 8 target OMPs in water matrices. No studies were found in the literature considering (non-polymeric) carbon materials in a multi-layer configuration inside SPE cartridges. The only three studies addressing multi-layer cartridges to extract OMPs (from water and olive oil) employ commercial materials developed by leading companies in this field: (i) graphitized carbon black (GCB, ENVI-Carb), polymeric weak anion exchanger (Oasis WAX) and polymeric weak cation exchanger (Oasis WCX)^46,47^; and (ii) zirconia-coated silica and C18^48^. A compromise between the SPE procedure optimized for CXs in the present work (relevant for metaflumizone) and the procedure optimized in our previous work for MWCNTs (that is not recommended for metaflumizone) was needed to obtain the best possible extraction efficiencies for all the 8 target compounds. On this basis, the SPE conditions selected for the next tests were 1000 mL of water sample at neutral pH (7.0 ± 0.1).

#### Optimization of the multi-layer configuration

**Figure 4:**
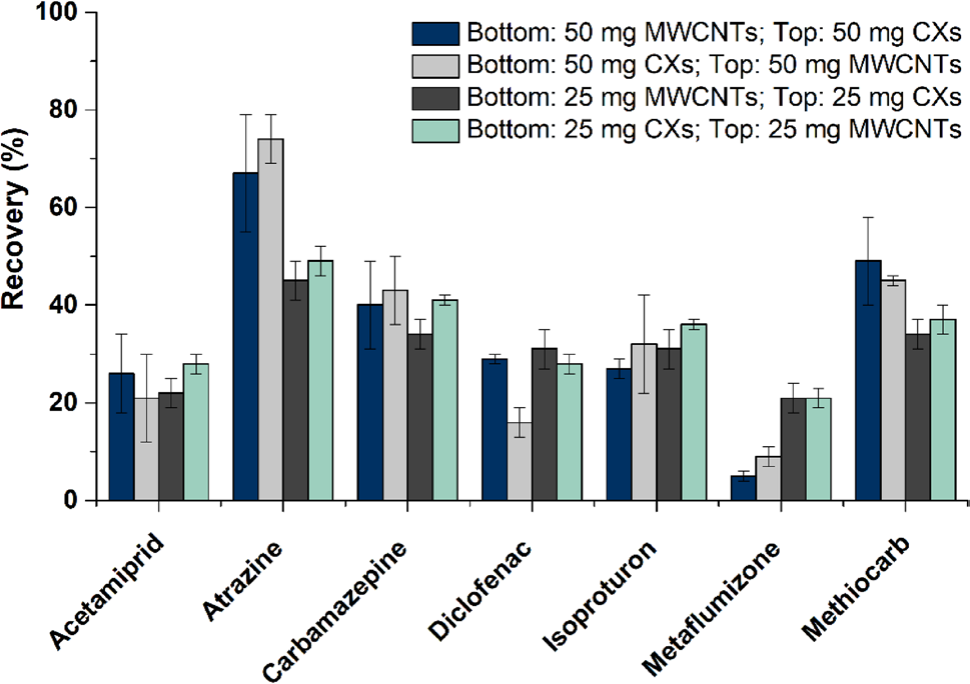
Recovery obtained for the target micropollutants (200 ng L^−1^ each), when using cartridges with different multi-layer configurations: type of carbon material (pristine CXs or MWCNTs) in each layer (bottom or top) and load of sorbents (25 and/or 50 mg). Experiments performed with 1000 mL of sample (SW; pH 7) and using ethanol as solvent (8 mL); *n* = 3 (RSD is represented as error bars).

The selected materials (pristine CXs and MWCNTs) were loaded in empty SPE cartridges, varying the multi-layer configuration, namely the type of carbon material in each layer (bottom and top) and load of sorbents (25 and/or 50 mg). The configurations tested and the respective results are shown in Fig. 4. As observed, the order of the carbon layers (i.e., CXs or MWCNTs loaded on the bottom or on the top of the cartridge) has no significant impact on the recoveries obtained. On the other hand, the quantity of sorbent loaded has some influence on the recoveries of the pesticides atrazine and methiocarb, for which a higher amount of carbon material provided a better performance; and metaflumizone, for which the recovery increases around 20% with the lower quantity of sorbent. Similar results were obtained for the other target OMPs regardless of the sorbent load, that may be explained by a more difficult desorption of this compound during the elution step, when higher amounts of CX were packed in the cartridges*.*

**Figure 5:**
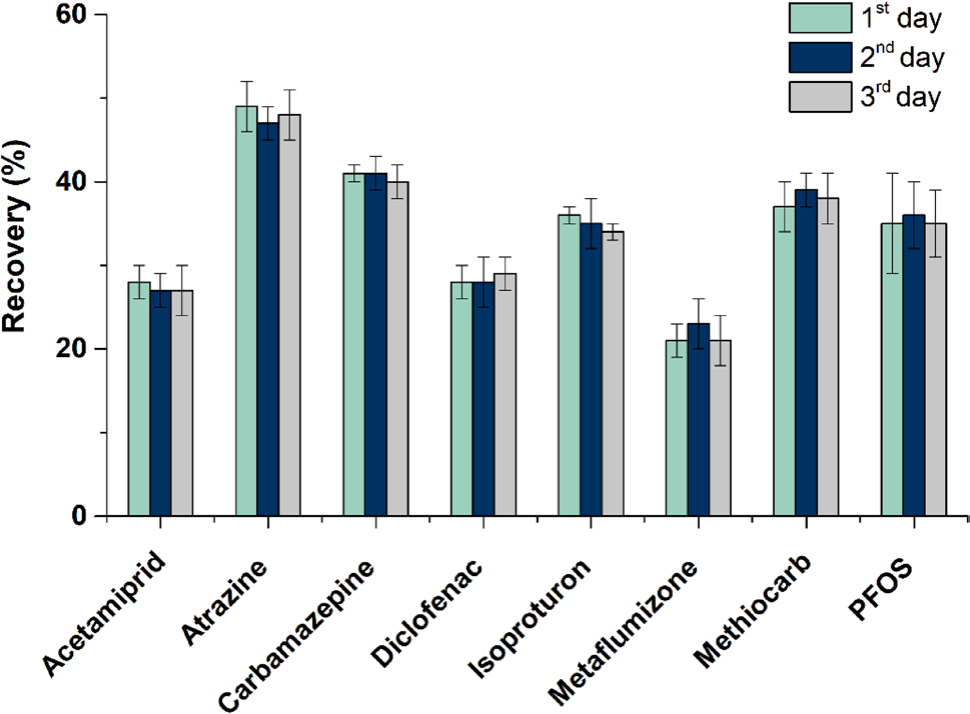
Recoveries obtained for micropollutants (200 ng L^−1^ each), extracting 1000 mL of water (pH 7) with 8 mL of ethanol as solvent, during consecutive reuse cycles performed with the same multi-layer carbon-based cartridge (bottom: 25 mg of CX; top: 25 mg of MWCNT); n = 3 (RSD is represented as error bars).

The recoveries obtained with the best multi-layer carbon-based cartridges particularly when considering metaflumizone (bottom: 25 mg of CXs; top: 25 mg of MWCNTs), were compared with those previously obtained with CXs and MWCNTs cartridges independently (Figure S8). A non-cumulative effect is observed, since the recoveries obtained when the two carbon materials are packed together in the same cartridge are nearly half of those obtained when CXs (in the case of metaflumizone) and MWCNTs cartridges (for the other 7 OMPs) are used independently. These results may be explained by the stronger adsorption on MWCNTs (in the case of metaflumizone) or on CXs (in the case of the other 7 OMPs), which hinders the desorption step (Table S9) and leads to a decrease in the recovery values (i.e., when comparing the performance of multi-layer cartridge with the single-layer cartridges packed with each carbon material). Only the multi-layer carbon-based SPE cartridge is able to extract in a single procedure the 8 target OMPs simultaneously. Although the recoveries are low (> 20%), the procedure was proved to be very precise (RSD < 6%). Additionally, in the literature, there is neither an official guideline nor a consensual recommendation for the recoveries that should be achieved during the development of an analytical methodology. Bearing this in mind and considering the constant recovery values for all analytes over the range of concentrations as well as the proportional response to the internal standards, it was decided to keep these analytes in this study. Therefore, the multi-layer carbon cartridge's reusability was studied, similar recoveries being obtained for the 8 OMPs during three successive cycles (Fig. 5). These reusability features are characteristic of carbon-based SPE cartridges^36^, representing an advantage when compared to traditional single-use commercial cartridges^1,49^. Moreover, this multi-layer cartridge is able to extract all the target analytes simultaneously, with a low load of sorbents and an eco-friendly solvent.

#### Application in a monitoring campaign focusing on SW and DW

The developed multi-layer carbon-based SPE cartridge was used to concentrate (preconcentration factor of 4000) and cleanup (Figure S1a,c) water samples prior to the analysis by UHPLC-MS/MS, in order to identify and quantify the target OMPs. A spatial monitoring program of SW collected from different Portuguese rivers was performed, providing a mapping on the occurrence of PSs and CECs in the environment that persist and might contaminate DW. For this purpose, the SW and DW samples were collected before and after 3 DWTPs, i.e., near the intake of each DWTP and at an endpoint of the distribution system served by that same plant, respectively. The results obtained are given in Table 2. As observed, 6 out of the 8 target compounds were detected in the collected water samples: atrazine, carbamazepine, diclofenac, isoproturon, metaflumizone, and methiocarb.

**Table 2:** Concentrations (ng L^−1^) of target micropollutants found in SW and DW near to the DWTP 1, 2 and 3.

Analyte	Concentration (ng L^−1^)					
	DWTP-1		DWTP-2		DWTP-3	
	SW-1	DW-1	SW-2	DW-2	SW-3	DW-3
Acetamiprid	n.d.^a^	n.d.	n.d.	n.d.	n.d.	n.d.
Atrazine	< MQL^b^	n.d.	n.d.	n.d.	198 ± 2	n.d.
Carbamazepine	83.8 ± 6.6	15.3 ± 2.4	69.6 ± 16.3	12.6 ± 2.9	68.5 ± 5.8	n.d.
Diclofenac	949 ± 24	31.8 ± 5.3	116 ± 5	n.d.	264 ± 3	n.d.
Isoproturon	168 ± 5	n.d.	131 ± 19	n.d.	339 ± 29	n.d.
Metaflumizone	n.d.	n.d.	159 ± 33	15.5 ± 0.4	72.9 ± 3.9	n.d.
Methiocarb	n.d.	n.d.	n.d.	n.d	138 ± 1	n.d.
PFOS	n.d.	n.d.	n.d.	n.d.	n.d.	n.d.

*n.d.* not detected, *MQL* method quantification limit.

Regarding the target pesticides of this study, the neonicotinoid acetamiprid was not detected. The banned triazine pesticide, atrazine, was quantified in SW-3 and detected below the MQL in SW-1. Isoproturon was quantified in the 3 SW samples analysed (up to 339 ± 29 ng L^−1^). Metaflumizone was quantified for the first time ever in both SW (SW-2 and SW-3) and DW (DW-2) samples. Methiocarb was found only once in SW-3 (138 ± 1 ng L^−1^). Moreover, the industrial compound PFOS was not detected in this study. In fact, the target compounds listed as PSs (atrazine, isoproturon and PFOS) were always quantified below their maximum allowable concentration-environmental quality standards (MAC-EQS), as defined in Directive 2013/39/EU for surface water bodies. Additionally, the sum of all the pesticides was below the maximum admissible concentration (i.e., 0.5 µg L^−1^) for this class of compounds in DW, as defined in Directive 98/83/EC.

The pharmaceutical compounds were detected at the highest concentrations and more frequently in both matrices (SW and DW), possibly due to their high prescription/usage nowadays. Carbamazepine, an anti-epileptic compound reported as recalcitrant in several monitoring studies^50–52^, was quantified up to 83.8 ± 6.6 ng L^−1^ in SW and 15.3 ± 2.4 ng L^−1^ in DW samples. The anti-inflammatory diclofenac was quantified at a high level in SW-1 (949 ± 24 ng L^−1^). This broadly consumed pharmaceutical was withdrawn from the most recent Watch List (EU Decision 2020/1161). Nevertheless, it is one of the compounds most frequently found and at highest concentrations in water bodies, being found even in DW samples^50–55^. These results suggest that neither the natural mechanisms occurring along the river course (i.e., photodegradation, biodegradation, among others), nor the dilution factor are sufficient to completely eliminate these pollutants that are released into the environment.

Summarizing, the results obtained allow concluding about the feasibility of employing this novel multi-layer carbon-based cartridge in a SPE-UHPLC–MS/MS method for monitoring PSs and CECs in SW and DW.

In addition, when comparing the studies published in the literature, dealing with the application of carbon materials in conventional SPE procedures (Table S10), it is possible to verify that the multi-layer carbon-based cartridge herein developed (i.e., with more than one type of carbon material) is the only one that has proved to extract, in a single procedure, metaflumizone and different classes of OMPs (pesticides, pharmaceutical and industrial compounds) listed in the abovementioned Directive^2^ and Decisions^3–5^.

## Conclusions

Pristine and HNO_3_ hydrothermally treated CXs were synthesized and used as SPE sorbents for the extraction of metaflumizone and other 7 multi-class OMPs from water matrices. The introduction of oxygenated functional groups by oxidation of the CX surface negatively affected the recoveries obtained for the target analytes, indicating that the adsorption/desorption process is more efficient on a less acidic surface. Overall, the recoveries obtained with pristine and functionalized CXs were low, except for metaflumizone (> 60%). Taking this into consideration, multi-layer carbon cartridges with pristine CXs and MWCNTs were tested as a proof of concept in the present work. The optimized cartridge configuration was able to extract the 8 target OMPs (with different p*K*a and polarity range) at once, using an eco-friendly solvent and low load of sorbents, and can be reused at least three times without affecting the extraction efficiency. Nevertheless, efforts should be done in the future with the purpose of improving the selectivity and specificity of the materials to achieve higher recovery values for the target compounds. Accordingly, the adsorption/desorption mechanisms of these 8 compounds over the carbon materials should be investigated in more detail. An analytical methodology based on SPE-UHPLC–MS/MS was then validated using the innovate multi-layer carbon-based cartridge. The potential of this method for monitoring EU-relevant OMPs was demonstrated through a monitoring campaign focusing on SW and DW samples collected before and after DWTPs, which confirmed the occurrence of a wide range of OMPs (at ng L^−1^ levels). Among the OMPs quantified (6 in total), the most commonly found was carbamazepine and diclofenac. Metaflumizone, a CEC recently added to the Watch List in EU Decision 2020/1161, was quantified in water courses for the first time, highlighting the importance of including this new CEC in future monitoring programs.

## Supplementary Information

Supplementary Information.
