# Supplementary material for: Carbon xerogels combined with nanotubes as solid-phase extraction sorbent to determine metaflumizone and seven other surface and drinking water micropollutants
Source: Sci Rep. 2021 Jul 5;11:13817. doi: 10.1038/s41598-021-93163-2 (PMC8257787; doi:10.1038/s41598-021-93163-2)
Supplement: Supplementary file 1 — Supplementary Information. [file 41598_2021_93163_MOESM1_ESM.docx]

**Supplementary Material**

**Carbon xerogels combined with nanotubes as solid-phase extraction sorbent to determine metaflumizone and seven other surface and drinking water micropollutants**

Marta O. Barbosa, Rui S. Ribeiro, Ana R.L. Ribeiro, M. Fernando R. Pereira, Adrián M.T. Silva*

Laboratory of Separation and Reaction Engineering - Laboratory of Catalysis and Materials (LSRE-LCM), Faculdade de Engenharia, Universidade do Porto, Rua Dr. Roberto Frias s/n, 4200-465 Porto, Portugal.

*Corresponding author: adrian@fe.up.pt (A.M.T. Silva)

**Supplementary Material Table of Contents**

**Text S1.** Conditions of chromatographic separation and MS parameters.

**Table S1.** Target compounds, class, structure, relative molecular mass (M_r_), p*K*a, and log K_OW_ values and solubility in water.

**Table S2.** Selected variables and respective levels investigated in the definitive screening design (DSD) and Box-Benkhen design (BBD); and quadratic polynomial model applied in BBD data evaluation.

**Table S3.** Selected reaction monitoring (SRM) instrument parameters for tandem mass spectrometry analysis of target analytes.

**Table S4.** Retention time, range, linearity, instrument and method detection, and quantification limits for each target analyte.

**Table S5.** Recovery, accuracy, precision (intra- and inter-batch), matrix effect and process efficiency for each target analyte with the developed multi-layer carbon-based cartridges; efficiency for each target analyte with the commercial cartridges Oasis HLB.

**Table S6.** Design of trial runs (in coded form) for DSD and corresponding recovery values for the 8 target compounds. Variables: (A) mass of sorbent material (mg); (B) sample volume (mL); (C) sample pH; (D) type of solvent; and (E) solvent volume (mL); All the experiments were performed with pristine CX samples.

**Table S7.** Design of trial runs (in coded form) for BBD and corresponding recovery values for the 8 target compounds. Variables: (A) mass of sorbent material (mg); (B) sample volume (mL); (C) sample pH; (D) type of solvent; and (E) solvent volume (mL); All the experiments were performed with pristine CX samples.

**Table S8.** ANOVA results of the quadratic regression model for optimization of the recovery efficiency (%) of metaflumizone.

**Table S9.** SPE recovery efficiencies (%) and batch desorption (%) obtained for the target compounds with: (i) original CX and MWCNT samples; and (ii) hydrothermally treated CX and MWCNT samples (0.3 mol L^-1^ of HNO_3_).

**Table S10.** Studies dealing with the application of carbon materials in conventional SPE (type of carbon-based material, target analytes and matrix) for the analysis of the PSs and CECs listed in Directive 2013 and Decisions 495/2015, 840/2018 and 1161/2020. Pollutants included in these studies that are out of the scope of EU legislation are not included in this table.

**Figure S1.** Total Ion Current (TIC) chromatograms of the 8 target OMPs (200 ng L^-1^) in: (a) a SPE extract of a spiked sample; (b) a post-spiked blank extract, using cartridges packed with multi-layer carbon-based cartridge (bottom: 25 mg of CXs; top: 25 mg of MWCNTs); and (c) an ethanolic standard. (This figure was produced using LabSolutions software Version 5.41 SP1, Shimadzu Corporation; <https://www.shimadzu.com/>).

**Figure S2.** Pareto charts representation of the standardized effects originated by the main factors (A, B, C, D and E) for each target compound; response is recovery (%); α = 0.05. All the experiments were performed with pristine CX samples.

**Figure S3.** Response surface and contour plots showing the effect of the mass of sorbent material (mg) (factor A), sample volume (mL) (factor B), and sample pH (factor C) on the recovery of metaflumizone: a) Response surface and contour plots of recovery as a function of factors A and B (hold value – C = 7); b) Response surface and contour plots of recovery as a function of factors A and C (hold value – B = 750 mL); c) Response surface and contour plots of recovery as a function of factors B and C (hold value – A= 100 mg). All the experiments were performed with pristine CX samples. (This figure was obtained using accomplished using Minitab Statistical Software, Minitab 19; <https://www.minitab.com/en-us/products/minitab/>).

**Figure S4.** N_2_ adsorption-desorption isotherms at -196 ^o^C of CXs subjected to hydrothermal treatment with different HNO_3_ concentrations: the subscript number in CX_0_, CX_0.01_, and CX_0.05_ corresponds to the concentration of HNO_3_ (mol L^-1^).

**Figure S5.** Recoveries obtained for the target micropollutants (200 ng L^-1^ each), when using cartridges packed with carbon xerogel (CX; 50 mg) obtained after hydrothermal treatment with different HNO_3_ concentrations (0-0.30 mol L^-1^). Experiments performed with 1000 mL of surface water (SW; pH 7) and using ethanol as solvent (8 mL); *n* = 3 (RSD is represented as error bars).

**Figure S6.** Recoveries obtained for the target micropollutants (200 ng L^-1^ each), when using cartridges packed with CXs (50 mg) with different solvents (ethanol + 5% of NH_4_OH, ethanol + 2% of CH_2_O_2_, and ethanol) and two elution steps (8 + 8 mL). Experiments performed with 1000 mL of SW (pH 7); *n* = 3 (RSD is represented as error bars).

**Figure S7.** Recovery obtained for metaflumizone as a function of ([CO_2_] + [CO])/S_BET_.

**Figure S8.** Recoveries obtained for the target micropollutants (200 ng L^-1^ each), when using cartridges packed with different carbon materials: multi-walled carbon nanotubes (MWCNTs), CXs and multi-layer (bottom: 25 mg of CXs; top: 25 mg of MWCNTs). Experiments performed with the optimized procedures for each type of sorbent; n = 3 (RSD is represented as error bars).

**Text S1.** Conditions of chromatographic separation and MS parameters

A column Kinetex™ XB-C18 100 Å (100 × 2.1 mm i.d.; particle diameter of 1.7 μm) purchased from Phenomenex, Inc. (Torrance, CA, USA) and operating under gradient mode of flow of the mobile phase water/ethanol (50/50, v/v) was used for the chromatographic separation of the target OMPs. The temperature of the column oven was set at 35 °C, the autosampler temperature at 15 °C, and the injection volume was 5 μL. Regarding the MS settings, these were defined to: argon at 230 kPa as CID gas, 400 °C and 250 °C for source and desolvation temperatures, capillary voltage of 0.5 kV, 2.5 dm^3^ min^-1^ and 12.5 dm^3^ min^-1^ of nebulizing gas flow and drying gas flow, respectively.

**Table S1.** Target compounds, class, structure, relative molecular mass (M_r_), p*K*a, and log *K*_OW_ values and solubility in water.

| **Compound** | **Class and sub class** | **Structure** | **M_r_** | **p*K*a** | **log *K*_OW_** | | **Solubility in water**  **(mg L^-1^)** |
| --- | --- | --- | --- | --- | --- | --- | --- |
| Acetamiprid ^a^ | Pesticide  *Neonicotinoid* | 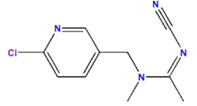 | 222.68 | 0.70 | 0.80 | 4250  (25 ºC) | |
| Atrazine ^b^ | Pesticide  *Triazine* | 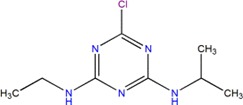 | 215.69 | 1.60 | 2.61 | 33.0  (25 ºC) | |
| Carbamazepine | Pharmaceutical  *Psychiatric drug* | 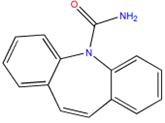 | 236.27 | 13.9 | 2.45 | 17.7  (25 ºC) | |
| Diclofenac ^c^ | Pharmaceutical  *Anti-inflammatory* | 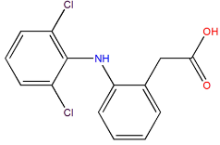 | 296.14 | 4.15 | 4.51 | 2.5  (25 ºC) | |
| Isoproturon ^b^ | Pesticide  *Phenylurea* | 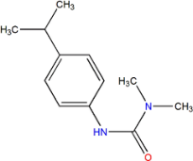 | 206.28 | n.a. | 2.87 | 70.0  (20 ºC) | |
| Metaflumizone ^a,d^ | Pesticide  *Insecticide* | 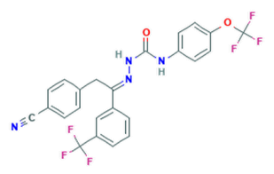 | 506.41 | n.a | n.a | n.a | |
| Methiocarb ^a^ | Pesticide  *Insecticide* | 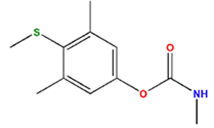 | 225.31 | 14.8 | 2.92 | 27.0  (20 ºC) | |
| Perfluorooctanesulfonic acid (PFOS) ^b^ | Industrial compound | 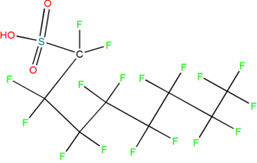 | 500.13 | -3.30 | 4.49 | 3.2×10^-3^  (25 ºC) | |

^a^ Contaminant of emerging concern of Decision 2018/840/EU; ^b^ Priority substance of Directive 2013/39/EU; ^c^ Contaminant of emerging concern of the former Decision 2015/495/EU; ^a^ Contaminant of emerging concern of Decision 2020/1161/EU; n.a. - not available.

**Table S2.** Selected variables and respective levels investigated in the definitive screening design (DSD) and Box-Benkhen design (BBD); and quadratic polynomial model applied in BBD data evaluation.

| **Definitive Screening Design (DSD)** | **Variables** | **Selected levels** | | |
| --- | --- | --- | --- | --- |
|  |  | -1 | 0 | +1 |
|  | (A) mass of sorbent material (mg) | 25 | 75 | 150 |
|  | (B) sample volume (mL) | 250 | 500 | 1000 |
|  | (C) sample pH | 3 | 7 | 11 |
|  | (D) type of solvent | methanol | - | ethanol |
|  | (E) solvent volume (mL) | 4 | 7 | 10 |
| **Box-Behnken Design (BBD)** | (A) mass of sorbent material (mg) | 50 | 100 | 150 |
|  | (B) sample volume (mL) | 500 | 750 | 1000 |
|  | (C) sample pH | 3 | 7 | 11 |
| **BBD**  **Quadratic polynomial model^a^** | 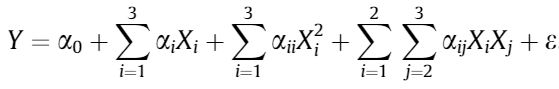 | | | Equation S1 |

^a^ Data from the BBD were analyzed by multiple regression to fit the following quadratic polynomial model (Equation S1), where:

Y is the response variable; α_0_ is the model constant; α_i_ is the linear coefficient; α_ii_ indicates the quadratic coefficient; α_ij_ is the interaction coefficient; and ε represents the statistical error.

**Table S3.** Selected reaction monitoring (SRM) instrument parameters for tandem mass spectrometry analysis of target analytes.

| **Analyte** | **IS set^a^** | **ESI mode (NI^b^ or PI^c^)** | **Precursor ion (m/z)** | **Quantification (SRM1)** | | | | **Confirmation (SRM2)** | | | |
| --- | --- | --- | --- | --- | --- | --- | --- | --- | --- | --- | --- |
|  |  |  |  | **Product Ion** | **DP^d^ (V)** | **CE^e^ (V)** | **CXP^f^ (V)** | **Product Ion** | **DP^d^ (V)** | **CE^e^ (V)** | **CXP^f^ (V)** |
| Acetamiprid | 1 | PI | 222.70 | 126.00 | -15 | -20 | -23 | 56.10 | -15 | -16 | -22 |
| Acetamiprid-d3 (1) | - | PI | 226.00 | 126.00 | -25 | -20 | -23 | - | - | - | - |
| Atrazine | 2 | PI | 216.00 | 174.00 | -24.0 | -16.0 | -30.0 | 68.05 | -24.0 | -36.0 | -10.0 |
| Atrazine-d5 (2) | - | PI | 221.10 | 179.05 | -11.0 | -18.0 | -17.0 | - | - | - | - |
| Carbamazepine | 3 | PI | 237.00 | 194.00 | -12.0 | -20.0 | -30.0 | 192.00 | -12.0 | -25.0 | -30.0 |
| Diclofenac | 3 | NI | 293.90 | 250.00 | 21.0 | 11.0 | 17.0 | 214.05 | 21.0 | 20.0 | 22.0 |
| Diclofenac-d4 (3) | - | NI | 297.95 | 254.05 | 21.0 | 12.0 | 28.0 | - | - | - | - |
| Isoproturon | 2 | PI | 206.80 | 72.00 | -15.0 | -20.0 | -29.0 | 46.00 | -15.0 | -18.0 | -16.0 |
| Metaflumizone | 4 | NI | 505.00 | 302.05 | 36.0 | 18.0 | 21.0 | 116.95 | 36.0 | 43.0 | 22.0 |
| Methiocarb | 4 | PI | 226.10 | 169.10 | -24.0 | -9.0 | -17.0 | 121.10 | -24.0 | -19.0 | -21.0 |
| Methiocarb-d3 (4) | - | PI | 229.10 | 169.10 | -25.0 | -11.0 | -30.0 | - | - | - | - |
| PFOS | 2 | NI | 498.70 | 79.95 | 18.0 | 50.0 | 14.0 | 99.00 | 18.0 | 46.0 | 18.0 |

^a^ IS is internal standard; ^b^ NI is negative ionization mode; ^c^ PI is positive ionization mode; ^d^ DP is the declustering potential; ^e^ CE is the collision energy; ^f^ CXP is the collision cell exit potential.

**Table S4.** Retention time, range, linearity, instrument and method detection, and quantification limits for each target analyte.

| **Analyte** | **Retention time**  **(min)** | **Range**  **(ng L^-1^)** | ***r*^2^** | **IDL^a^**  **(µg L^-1^)** | **IQL^b^**  **(µg L^-1^)** | **MDL^c^**  **(ng L^-1^)** | **MQL^d^**  **(ng L^-1^)** |
| --- | --- | --- | --- | --- | --- | --- | --- |
| Acetamiprid | 1.06 | 36.1 – 400 | 0.999 | 283 | 857 | 11.9 | 36.1 |
| Atrazine | 1.94 | 13.8 – 400 | 0.998 | 46.3 | 140 | 4.55 | 13.8 |
| Carbamazepine | 1.59 | 7.86 – 400 | 0.999 | 27.7 | 83.9 | 2.59 | 7.86 |
| Diclofenac | 2.67 | 5.54 – 400 | 0.999 | 31.7 | 96.2 | 1.83 | 5.54 |
| Isoproturon | 2.18 | 15.5 – 400 | 0.998 | 79.2 | 240 | 5.13 | 15.5 |
| Metaflumizone | 27.9 | 13.3 – 400 | 0.996 | 134 | 423 | 4.37 | 13.3 |
| Methiocarb | 2.73 | 53.5 – 400 | 0.999 | 255 | 771 | 17.6 | 53.5 |
| PFOS | 1.88 | 4.83 – 400 | 0.997 | 16.4 | 49.7 | 1.59 | 4.83 |

^a^ IDL is instrument detection limit; ^b^ IQL is instrument quantification limit; ^c^ MDL is method detection limit; ^d^ MQL is method quantification limit.

**Table S5.** Recovery, accuracy, precision (intra- and inter-batch), matrix effect and process efficiency for each target analyte with the developed multi-layer carbon-based cartridges; efficiency for each target analyte with the commercial cartridges Oasis HLB.

| **Analyte** | **Recovery**  **(%)** | **Accuracy**  **(%)** | **Intra-batch precision**  **RSD (%)** | **Inter-batch precision**  **RSD (%)** | **Matrix effect**  **(%)** | **Process efficiency**  **(%)** | **Commercial cartridges:**  **Process efficiency (%)** |
| --- | --- | --- | --- | --- | --- | --- | --- |
| Acetamiprid | 27.3 | 96.5 ± 12.3 | 3.62 – 7.61 | 7.68 | -38.4 | 16.8 | 12.6 |
| Atrazine | 52.0 | 92.5 ± 9.4 | 10.2 – 11.3 | 13.4 | -24.4 | 39.3 | 34.6 |
| Carbamazepine | 40.6 | 86.2 ± 6.3 | 6.03 – 7.65 | 11.8 | -7.6 | 37.5 | 85.3 |
| Diclofenac | 31.7 | 106 ± 5 | 2.87 – 3.87 | 4.60 | -27.4 | 23.0 | 66.4 |
| Isoproturon | 34.6 | 83.4 ± 9.5 | 6.30 – 6.54 | 8.33 | -25.2 | 25.9 | 83.3 |
| Metaflumizone | 20.5 | 101 ± 7 | 7.86 – 9.26 | 11.2 | -38.8 | 12.6 | 44.1 |
| Methiocarb | 47.1 | 100 ± 6 | 5.31 – 6.04 | 5.81 | -41.2 | 27.7 | 142 |
| PFOS | 34.3 | 84.3 ± 9.0 | 7.48 – 11.3 | 14.5 | 13.5 | 38.9 | 30.4 |

**Table S6.** Design of trial runs (in coded form) for DSD and corresponding recovery values for the 8 target compounds. Variables: (A) mass of sorbent material (mg); (B) sample volume (mL); (C) sample pH; (D) type of solvent; and (E) solvent volume (mL); All the experiments were performed with pristine CX samples.

| **Experimental number** | **Variables** | | | | | **Recovery (%)** | | | | | | | |
| --- | --- | --- | --- | --- | --- | --- | --- | --- | --- | --- | --- | --- | --- |
|  | **A** | **B** | **C** | **D** | **E** | **Atrazine** | **Carbamazepine** | **Acetamiprid** | **Isoproturon** | **Methiocarb** | **Diclofenac** | **PFOS** | **Metaflumizone** |
| 1 | + | - | - | + | 0 | 9 | 6 | 2 | 6 | 7 | 7 | 2 | 1 |
| 2 | + | + | - | - | + | 29 | 29 | 0 | 21 | 19 | 23 | 11 | 8 |
| 3 | + | - | + | - | + | 11 | 8 | 7 | 6 | 0 | 2 | 13 | 5 |
| 4 | 0 | 0 | 0 | + | 0 | 15 | 16 | 7 | 9 | 6 | 3 | 8 | 20 |
| 5 | + | 0 | + | - | - | 13 | 9 | 6 | 6 | 0 | 2 | 23 | 5 |
| 6 | 0 | + | + | + | + | 18 | 10 | 0 | 7 | 3 | 3 | 27 | 54 |
| 7 | - | + | + | - | 0 | 4 | 3 | 3 | 4 | 0 | 1 | 10 | 60 |
| 8 | - | + | - | + | - | 1 | 0 | 0 | 2 | 0 | 2 | 1 | 55 |
| 9 | - | - | + | + | - | 1 | 1 | 0 | 0 | 0 | 0 | 2 | 17 |
| 10 | + | + | 0 | + | - | 18 | 13 | 11 | 8 | 6 | 3 | 15 | 36 |
| 11 | 0 | - | - | - | - | 7 | 9 | 0 | 8 | 5 | 2 | 1 | 1 |
| 12 | - | 0 | - | + | + | 9 | 4 | 3 | 12 | 2 | 4 | 2 | 4 |
| 13 | - | - | 0 | - | + | 7 | 7 | 5 | 3 | 1 | 3 | 5 | 22 |
| 14 | 0 | 0 | 0 | - | 0 | 13 | 13 | 14 | 8 | 4 | 2 | 10 | 26 |

| **Experimental number** | **Variables** | | | **Recovery (%)** | | | | | | | |
| --- | --- | --- | --- | --- | --- | --- | --- | --- | --- | --- | --- |
|  | **A** | **B** | **C** | **Atrazine** | **Carbamazepine** | **Acetamiprid** | **Isoproturon** | **Methiocarb** | **Diclofenac** | **PFOS** | **Metaflumizone** |
| 1 | 0 | - | + | 15 | 6 | 5 | 6 | 0 | 4 | 17 | 13 |
| 2 | + | 0 | - | 20 | 18 | 5 | 11 | 17 | 13 | 3 | 16 |
| 3 | 0 | + | + | 23 | 11 | 0 | 10 | 0 | 9 | 34 | 28 |
| 4 | + | - | 0 | 41 | 28 | 13 | 18 | 6 | 2 | 7 | 8 |
| 5 | - | + | 0 | 14 | 8 | 5 | 7 | 5 | 3 | 5 | 69 |
| 6 | 0 | - | - | 9 | 9 | 3 | 8 | 12 | 8 | 2 | 7 |
| 7 | - | 0 | - | 9 | 7 | 4 | 6 | 5 | 7 | 1 | 28 |
| 8 | - | - | 0 | 6 | 5 | 3 | 3 | 3 | 1 | 4 | 47 |
| 9 | 0 | 0 | 0 | 14 | 8 | 5 | 6 | 5 | 3 | 8 | 22 |
| 10 | 0 | 0 | 0 | 15 | 9 | 5 | 6 | 6 | 4 | 9 | 23 |
| 11 | - | 0 | + | 12 | 11 | 5 | 8 | 0 | 4 | 15 | 62 |
| 12 | + | + | 0 | 39 | 23 | 10 | 19 | 10 | 3 | 10 | 25 |
| 13 | 0 | 0 | 0 | 26 | 18 | 8 | 14 | 8 | 3 | 9 | 21 |
| 14 | 0 | + | - | 11 | 12 | 5 | 17 | 32 | 14 | 5 | 12 |
| 15 | + | 0 | + | 36 | 15 | 9 | 15 | 0 | 6 | 41 | 15 |

**Table S7.** Design of trial runs (in coded form) for BBD and corresponding recovery values for the 8 target compounds. Variables: (A) mass of sorbent material (mg); (B) sample volume (mL); (C) sample pH; (D) type of solvent; and (E) solvent volume (mL); All the experiments were performed with pristine CX samples.

**Table S8.** ANOVA results of the quadratic regression model for optimization of the recovery efficiency (%) of metaflumizone.

| **Source of variations** | **Sum of squares** | **Degree of freedom** | **Mean square** | **F-value** | **p-value** | **Determination coefficient (R^2^)** | **Adjusted determination coefficient (Adj R^2^)** |
| --- | --- | --- | --- | --- | --- | --- | --- |
| Model | 4760.00 | 9 | 528.89 | 21.27 | 0.002^a^ | 97.45% | 92.87% |
| A | 2491.60 | 1 | 2491.60 | 100.19 | 0.000 |  |  |
| B | 432.44 | 1 | 432.44 | 17.39 | 0.009 |  |  |
| C | 368.43 | 1 | 368.43 | 14.82 | 0.012 |  |  |
| A*A | 866.56 | 1 | 866.56 | 34.85 | 0.002 |  |  |
| B*B | 0.00 | 1 | 0.00 | 0.00 | 0.995 |  |  |
| C*C | 183.02 | 1 | 183.02 | 7.36 | 0.042 |  |  |
| A*B | 5.50 | 1 | 5.50 | 0.22 | 0.658 |  |  |
| A*C | 317.85 | 1 | 317.85 | 12.78 | 0.016 |  |  |
| B*C | 25.35 | 1 | 25.35 | 1.02 | 0.359 |  |  |
| Residual | 124.34 | 5 | 24.87 |  |  |  |  |
| Lack-of-fit | 122.24 | 3 | 40.75 | 38.77 | 0.025 |  |  |
| Pure error | 2.10 | 2 | 1.05 |  |  |  |  |
| Total | 4884.34 | 14 | 528.89 | 21.27 | 0.002 |  |  |
| Regression equation (uncoded unit) | Recovery efficiency (%) = 37,8 - 1,196 A + 0,0215 B + 10,43 C + 0,00613 A*A - 0,000000 B*B - 0,440 C*C - 0,000094 A*B - 0,0446 A*C + 0,00252 B*C | | | | | | |

^a^ Significant at 5% level.

**Table S9.** SPE recovery efficiencies (%) and batch desorption (%) obtained for the target compounds with: (i) original CX and MWCNT samples; and (ii) hydrothermally treated CX and MWCNT samples (0.3 mol L^-1^ of HNO_3_).

| **Target Compounds** | **Original CX** | | **CX - 0.3 mol L^-1^ of HNO_3_** | | **Original MWCNT** | | **MWCNT - 0.3 mol L^-1^ of HNO_3_** | |
| --- | --- | --- | --- | --- | --- | --- | --- | --- |
|  | **SPE Recovery Efficiency (%)** | **Desorption (%)** | **SPE Recovery Efficiency (%)** | **Desorption (%)** | **SPE Recovery Efficiency (%)** | **Desorption (%)** | **SPE Recovery Efficiency (%)** | **Desorption (%)** |
| Acetamiprid | 5 | 9 | 0 | 21 | 63 | 59 | 42 | 36 |
| Atrazine | 14 | 21 | 11 | 38 | 84 | 87 | 86 | 41 |
| Carbamazepine | 8 | 30 | 8 | 32 | 82 | 72 | 70 | 67 |
| Diclofenac | 3 | 7 | 2 | 4 | 65 | 62 | 47 | 16 |
| Isoproturon | 7 | 17 | 13 | 45 | 77 | 70 | 76 | 56 |
| Metaflumizone | 69 | 54 | 40 | 31 | 2 | 0 | 6 | 4 |
| Methiocarb | 5 | 8 | 0 | 20 | 53 | 62 | 75 | 74 |
| PFOS | 18 | 53 | 9 | 41 | 50 | 44 | 77 | 64 |

**Table S10.** Studies dealing with the application of carbon materials in conventional SPE (type of carbon-based material, target analytes and matrix) for the analysis of the PSs and CECs listed in Directive 2013 and Decisions 495/2015, 840/2018 and 1161/2020. Pollutants included in these studies that are out of the scope of EU legislation are not included in this table.

| **Carbon-based material** | | **Target analytes** | | **Matrix** | **Ref.** | | |
| --- | --- | --- | --- | --- | --- | --- | --- |
| **Multi-walled carbon nanotubes**  **(MWCNTs)** | | Atrazine and simazine | | Tap and groundwater | [1] | | |
|  |  | Atrazine and metabolites | | Surface and underground water | [2] | | |
|  |  | Atrazine and simazine | | River water | [3] | | |
|  |  | Atrazine and simazine | | River, tap, reservoir and wastewater | [4] | | |
|  |  | Atrazine | | Tap, reservoir and stream water | [5] | | |
|  |  | Chlorpyrifos | | Mineral water, groundwater, and run-off water from an agricultural | [6] | | |
|  |  | Chlorpyrifos | | Well, tap and river water | [7] | | |
|  |  | Atrazine | | Tap and reservoir water | [8] | | |
|  |  | Methiocarb | | Tap and surface water | [9] | | |
|  |  | Alachlor | | Tap and river water | [10] | | |
|  |  | Thiamethoxam, acetamiprid, and imidacloprid | | Tap, ground and  reservoir water | [11] | | |
|  |  | Diclofenac | | Surface and tap water | [12] | | |
|  |  | PFOS | | Tap and river water | [13] | | |
|  |  | PCP | | River water | [14] | | |
|  |  | PCP | | Tap and river water | [15] | | |
|  |  | PAHs | | Tap, river and seawater | [16] | | |
|  |  | PAHs | | River, tap and wastewater | [17] | | |
|  |  | Diclofenac | | River water | [18] | | |
|  |  | Atrazine and simazine | | River, tap and wastewater | [19] | | |
|  |  | 17-beta-estradiol | | Lake water | [20] | | |
|  |  | Erythromycin, azithromycin, and diclofenac | | Surface and groundwater | [21] | | |
|  |  | Acetamiprid, atrazine, diclofenac, isoproturon, metaflumizone*, methiocarb, PFOS | | Surface water | [22] | | |
| **Graphene-based materials:**  **graphene, graphene oxide, graphene aerogel** | | PCP | | Environmental water | [23] | | |
|  |  | PCP | | Pond and lake water | [24] | | |
|  |  | PAHs | | Rain, river and tap water | [25] | | |
|  |  | PAHs | | Pond and river water | [26] | | |
|  |  | PAHs | | Well, tap river and wastewater | [27] | | |
|  |  | Chlorfenvinphos | | Water | [28] | | |
|  |  | Diclofenac | | Wastewater | [29] | | |
|  |  | Ciprofloxacin | | Tap and river water | [30] | | |
|  |  | Estrone, 17-beta-estradiol, estriol, and 17-alpha-ethynylestradiol | | Tap and river water | [31] | | |
| **Carbon nanocones/**  **disks** | PCP | | Drinking, swimming pool, well and tank water | | | [32] |  |
| **Fullerene C_60_** | | Benzene | | Drinking, river, rain, ground and wastewater | [33] | | |
| **Asphalt-derived porous activated carbon** | | PAHs | | Tap water | [34] | | |
| **Multi-layer carbon-based cartridge: MWCNTs and Carbon Xerogels (CXs)** | | Acetamiprid, atrazine, diclofenac, isoproturon, metaflumizone, methiocarb, PFOS | | Surface and drinking water | **This work** | | |

n.a. is not available; PFOS is perfluorooctanesulfonic acid; PAHs is polycyclic aromatic hydrocarbons; PCP is pentachlorophenol; *the cartridge with MWCNTs only was ineffective for metaflumizone.


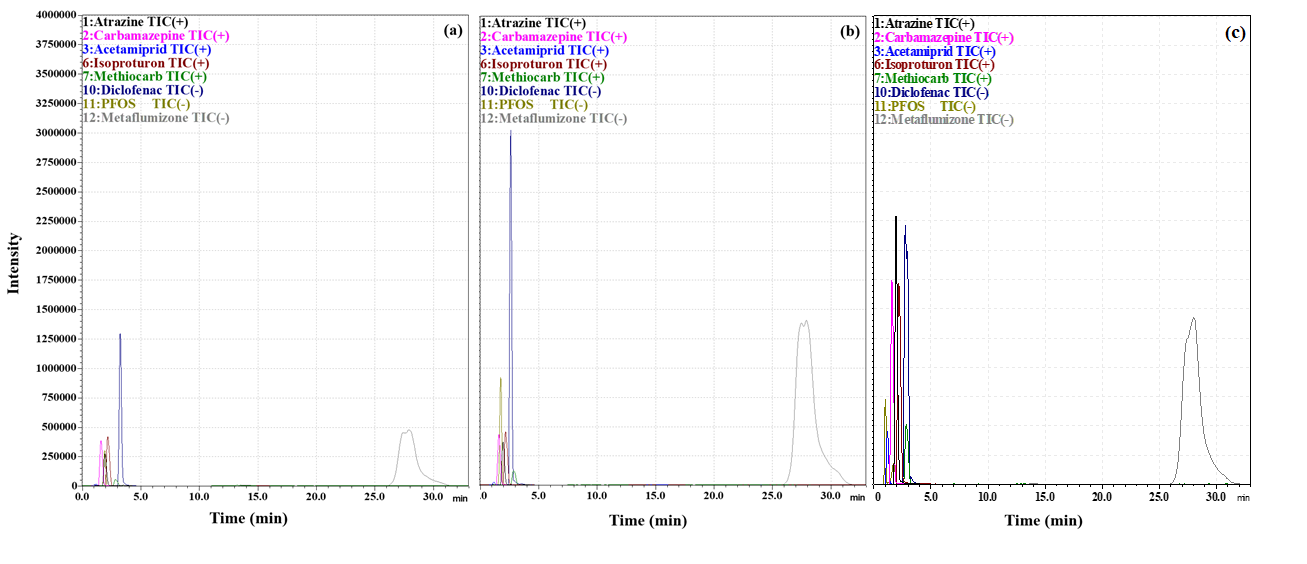


**Figure S1.** Total Ion Current (TIC) chromatograms of the 8 target OMPs (200 ng L^-1^) in: (a) a SPE extract of a spiked sample; (b) a post-spiked blank extract, using cartridges packed with multi-layer carbon-based cartridge (bottom: 25 mg of CXs; top: 25 mg of MWCNTs); and (c) an ethanolic standard. (This figure was produced using LabSolutions software Version 5.41 SP1, Shimadzu Corporation; <https://www.shimadzu.com/>).


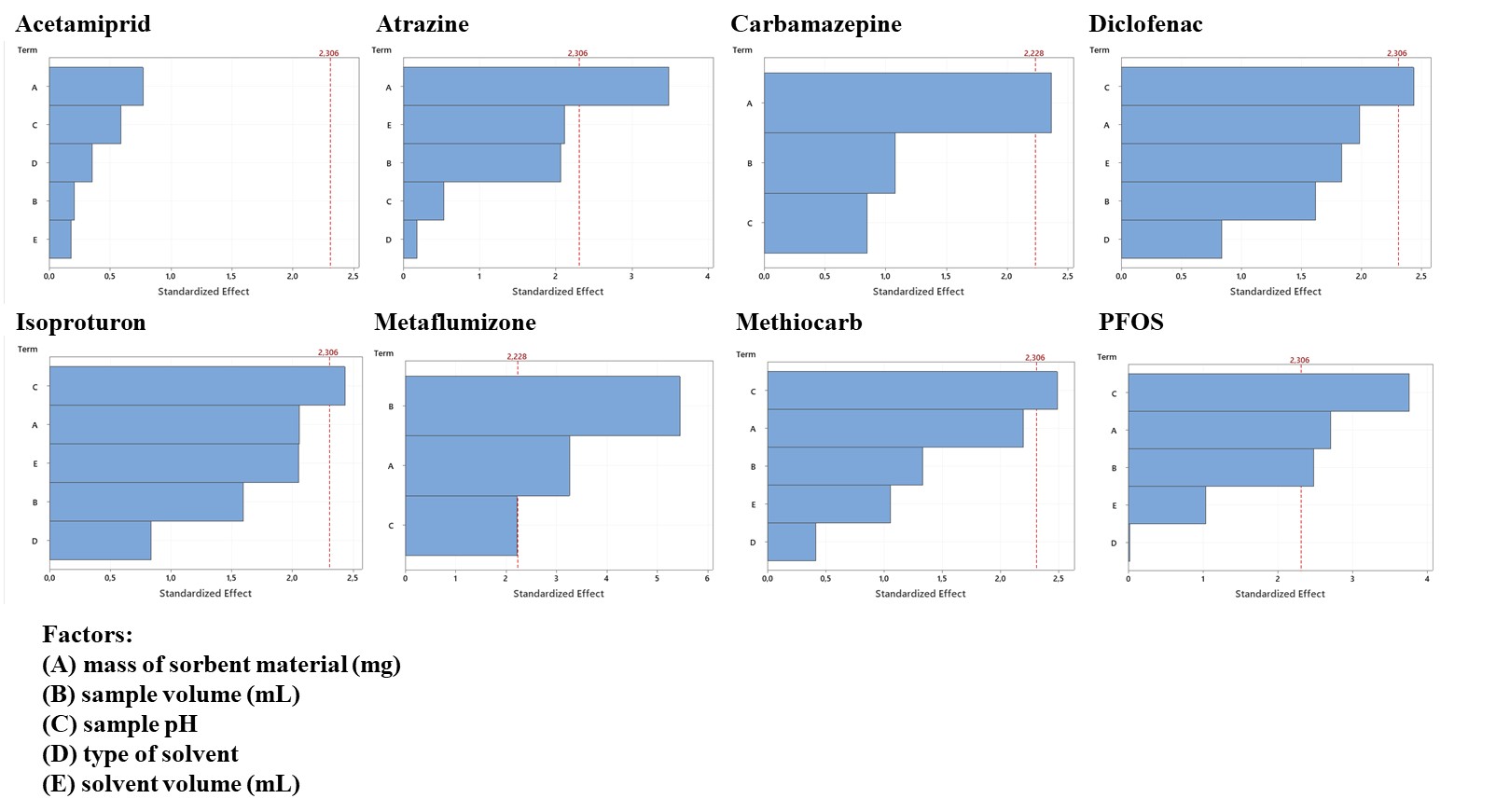


**Figure S2.** Pareto charts representation of the standardized effects originated by the main factors (A, B, C, D and E) for each target compound; response is recovery (%); α = 0.05. All the experiments were performed with pristine CX samples.


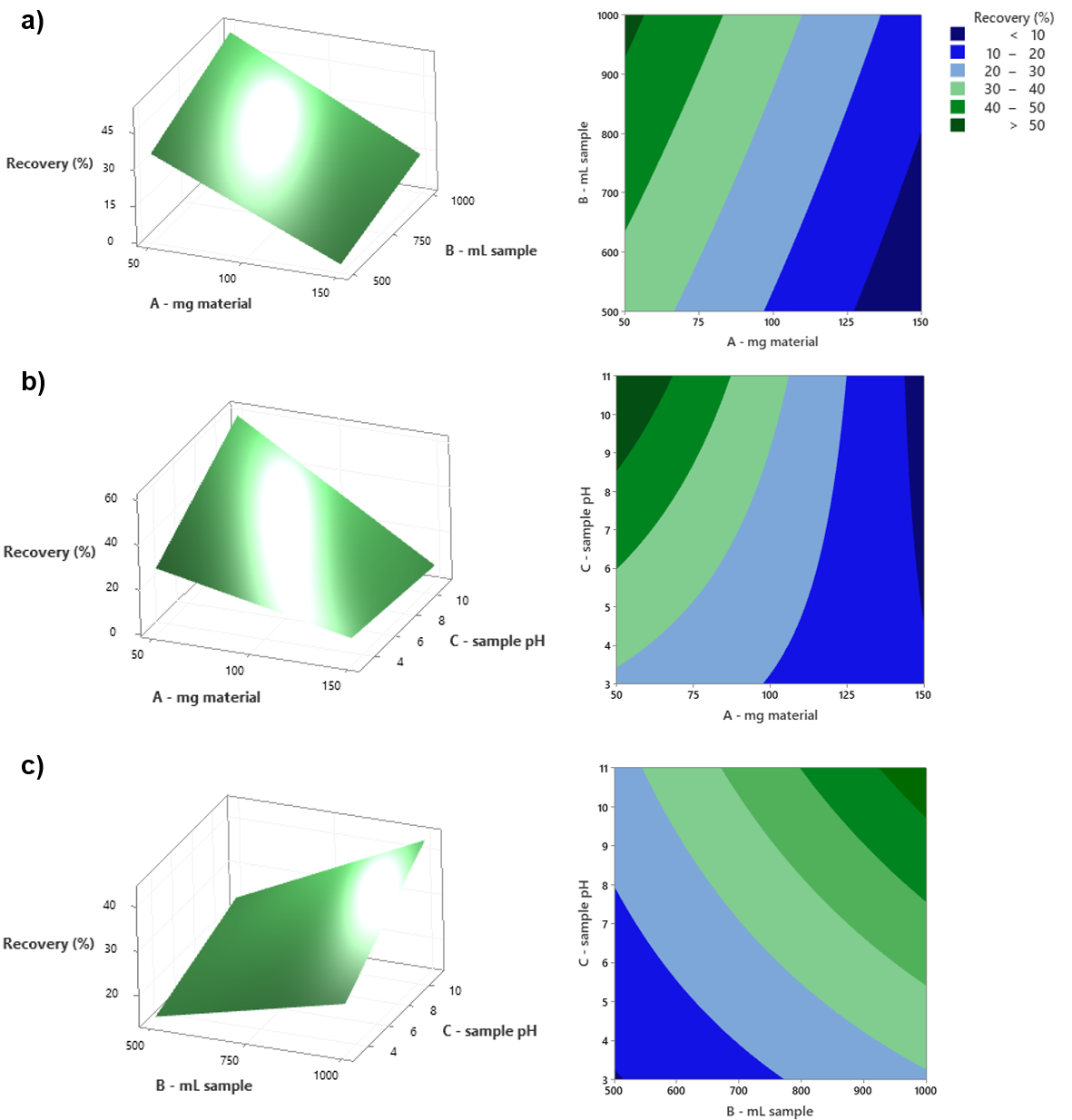


**Figure S3.** Response surface and contour plots showing the effect of the mass of sorbent material (mg) (factor A), sample volume (mL) (factor B), and sample pH (factor C) on the recovery of metaflumizone: a) Response surface and contour plots of recovery as a function of factors A and B (hold value: C = 7); b) Response surface and contour plots of recovery as a function of factors A and C (hold value: B = 750 mL); c) Response surface and contour plots of recovery as a function of factors B and C (hold value: A = 100 mg). All the experiments were performed with pristine CX samples. (This figure was obtained using accomplished using Minitab Statistical Software, Minitab 19; <https://www.minitab.com/en-us/products/minitab/>).


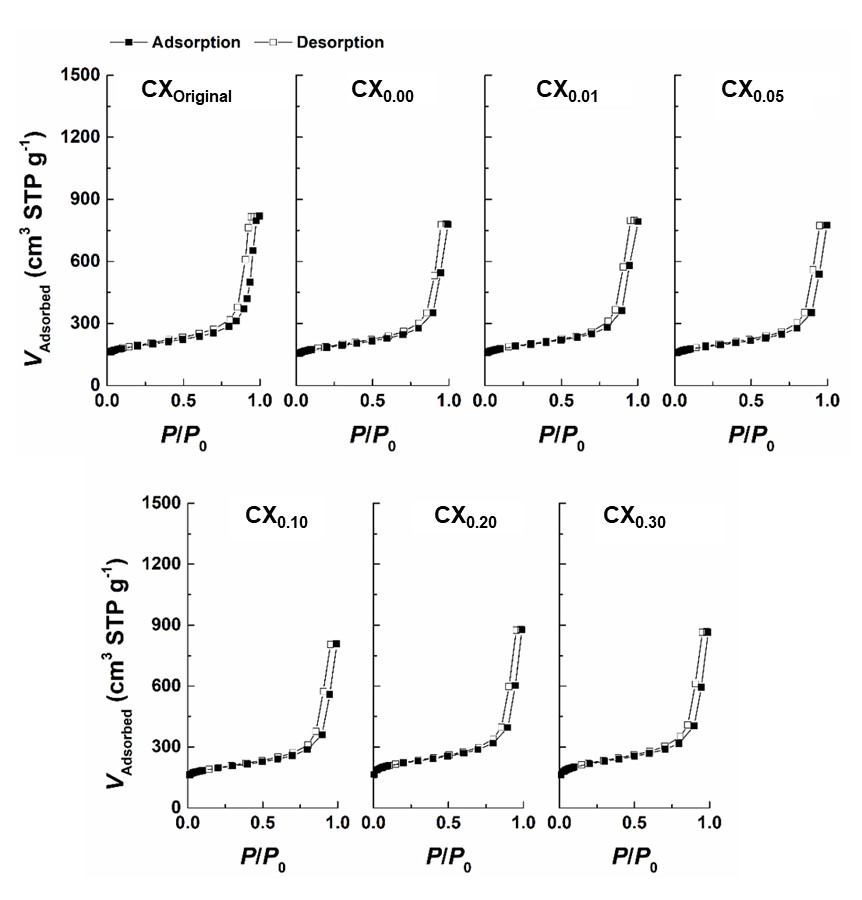


**Figure S4.** N_2_ adsorption-desorption isotherms at -196 ^o^C of CXs subjected to hydrothermal treatment

with different HNO_3_ concentrations: the subscript number in CX_0_, CX_0.01_, and CX_0.05_ corresponds to the concentration of HNO_3_ (mol L^-1^).


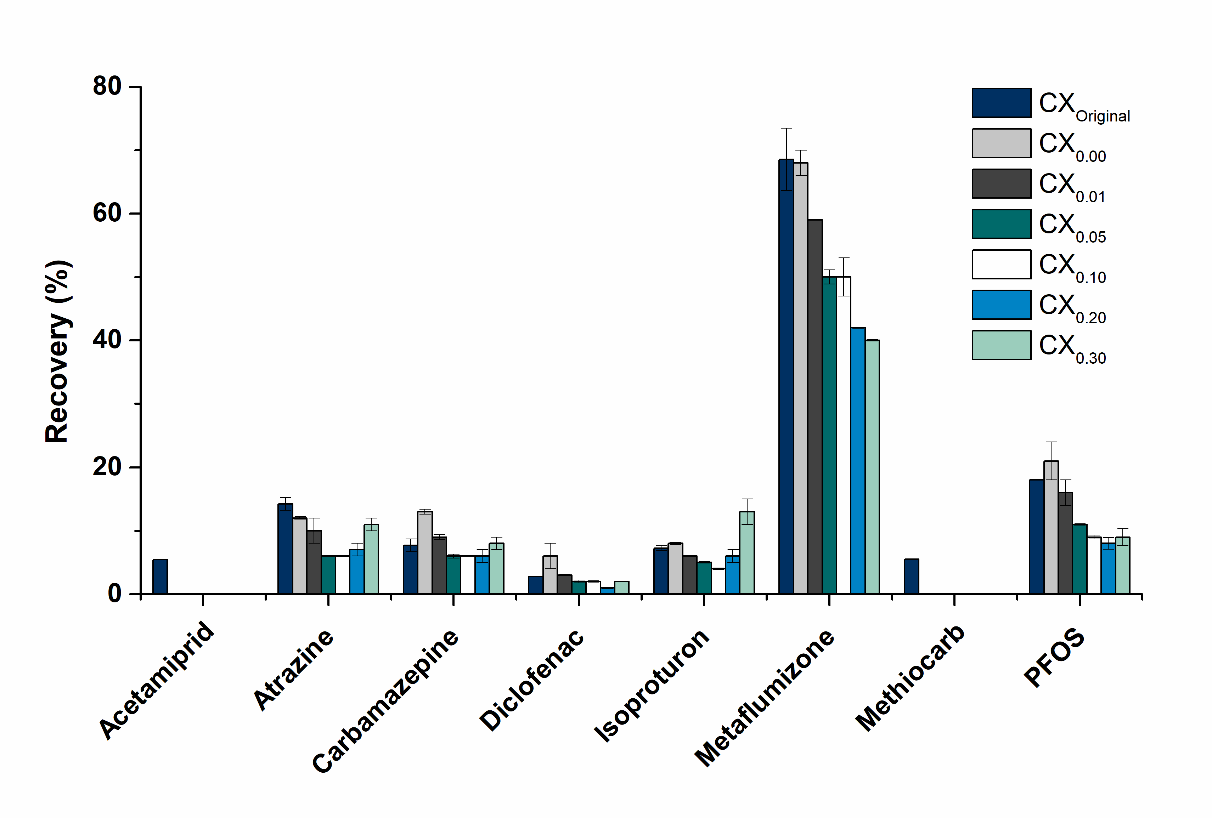


**Figure S5.** Recoveries obtained for the target micropollutants (200 ng L^-1^ each), when using cartridges packed with carbon xerogel (CX; 50 mg) obtained after hydrothermal treatment with different HNO_3_ concentrations (0-0.30 mol L^-1^). Experiments performed with 1000 mL of surface water (SW; pH 7) and using ethanol as solvent (8 mL); n = 3 (RSD is represented as error bars).


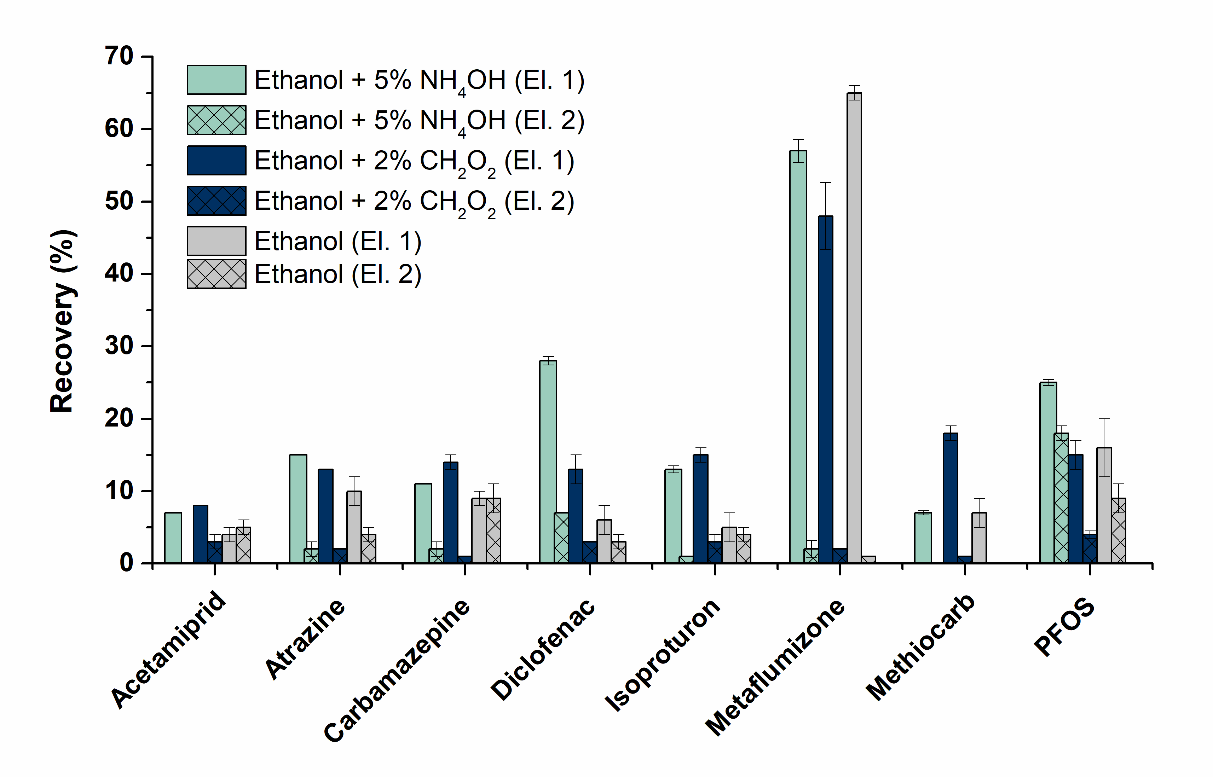


**Figure S6.** Recoveries obtained for the target micropollutants (200 ng L^-1^ each), when using cartridges packed with CXs (50 mg) with different solvents (ethanol + 5% of NH_4_OH, ethanol + 2% of CH_2_O_2_, and ethanol) and two elution steps (8 + 8 mL). Experiments performed with 1000 mL of SW (pH 7); .n = 3 (RSD is represented as error bars).


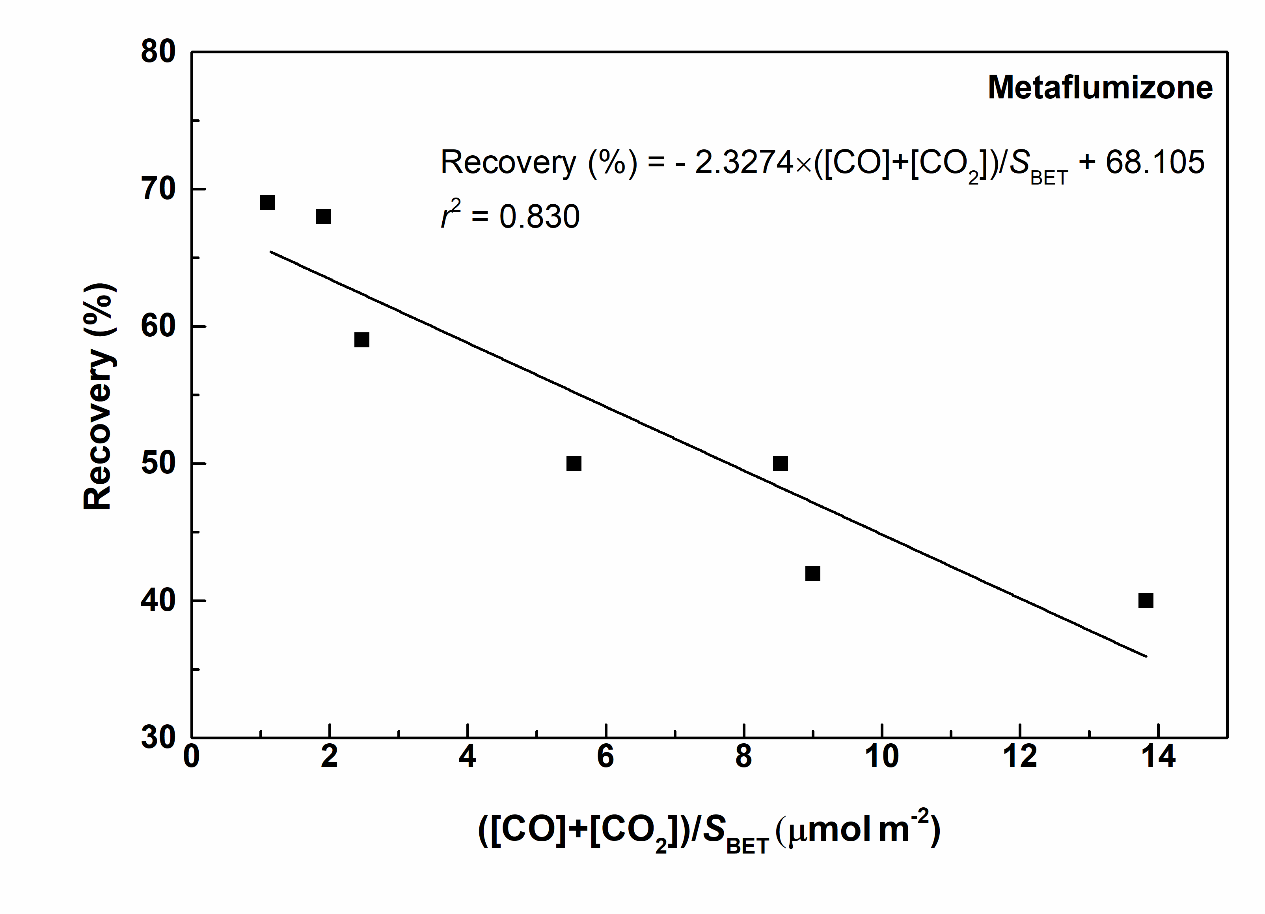


**Figure S7.** Recovery obtained for metaflumizone as a function of ([CO_2_] + [CO])/S_BET_.


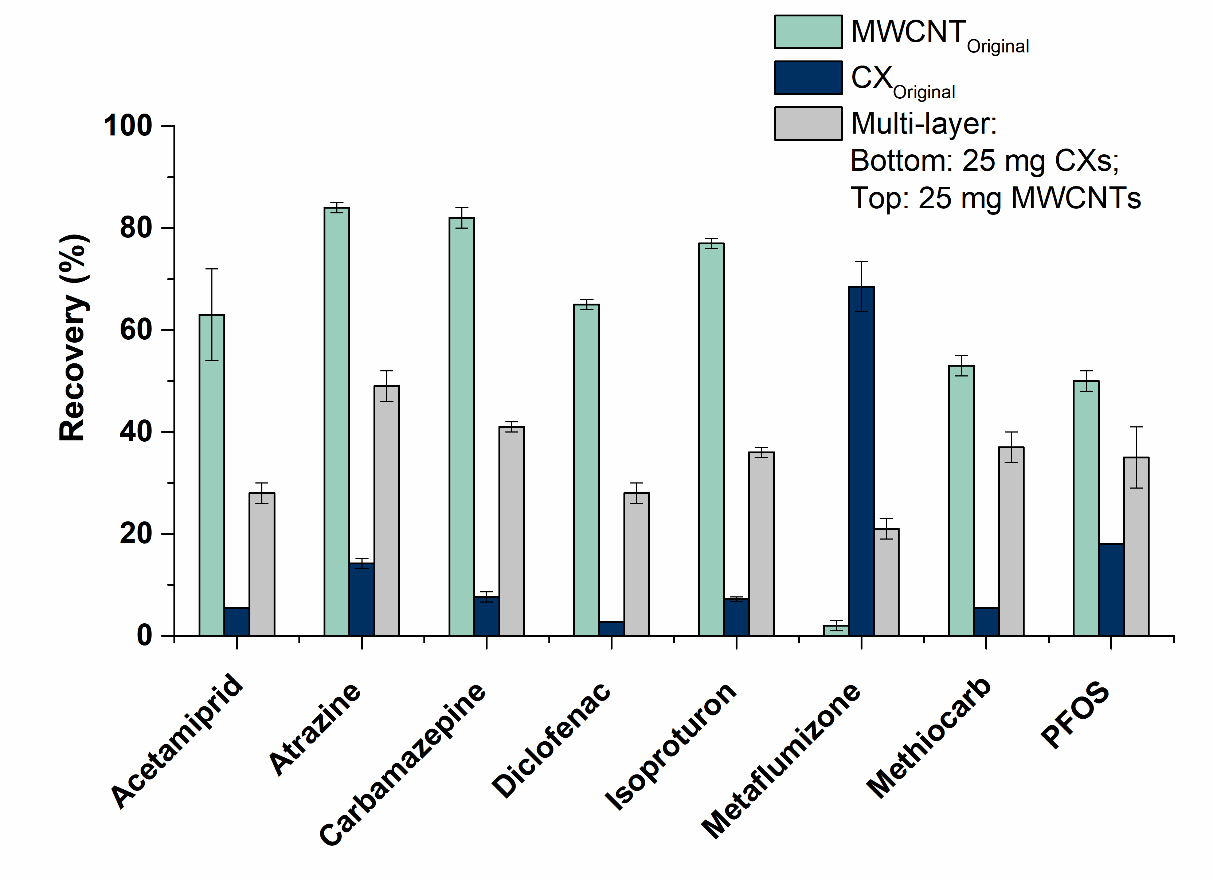


**Figure S8.** Recoveries obtained for the target micropollutants (200 ng L^-1^ each), when using cartridges packed with different carbon materials: multi-walled carbon nanotubes (MWCNTs), CXs and multi-layer (bottom: 25 mg CXs; top: 25 mg MWCNTs). Experiments performed with the optimized procedures for each type of sorbent; *n* = 3 (RSD is represented as error bars).

**References**

[1] Y.S. Al-Degs, M.A. Al-Ghouti, Preconcentration and determination of high leachable pesticides residues in water using solid-phase extraction coupled with high-performance liquid chromatography, International Journal of Environmental Analytical Chemistry, 88 (2008) 487-498.

[2] G. Min, S. Wang, H. Zhu, G. Fang, Y. Zhang, Multi-walled carbon nanotubes as solid-phase extraction adsorbents for determination of atrazine and its principal metabolites in water and soil samples by gas chromatography-mass spectrometry, Science of The Total Environment, 396 (2008) 79-85.

[3] Z.-g. Yu, Z. Qin, H.-r. Ji, X. Du, Y.-h. Chen, P. Pan, H. Wang, Y.-y. Liu, Application of SPE Using Multi-Walled Carbon Nanotubes as Adsorbent and Rapid Resolution LC-MS-MS for the Simultaneous Determination of 11 Triazine Herbicides Residues in River Water, Chromatographia, 72 (2010) 1073-1081.

[4] Q. Zhou, J. Xiao, W. Wang, G. Liu, Q. Shi, J. Wang, Determination of atrazine and simazine in environmental water samples using multiwalled carbon nanotubes as the adsorbents for preconcentration prior to high performance liquid chromatography with diode array detector, Talanta, 68 (2006) 1309-1315.

[5] A.H. El-Sheikh, J.A. Sweileh, Y.S. Al-Degs, A.A. Insisi, N. Al-Rabady, Critical evaluation and comparison of enrichment efficiency of multi-walled carbon nanotubes, C18 silica and activated carbon towards some pesticides from environmental waters, Talanta, 74 (2008) 1675-1680.

[6] L.M. Ravelo-Pérez, J. Hernández-Borges, M. Ángel Rodríguez-Delgado, Multiwalled carbon nanotubes as solid-phase extraction materials for the gas chromatographic determination of organophosphorus pesticides in waters, Journal of Separation Science, 31 (2008) 3612-3619.

[7] M.R. Hadjmohammadi, M. Peyrovi, P. Biparva, Comparison of C18 silica and multi-walled carbon nanotubes as the adsorbents for the solid-phase extraction of Chlorpyrifos and Phosalone in water samples using HPLC, Journal of Separation Science, 33 (2010) 1044-1051.

[8] Y.S. Al-Degs, M.A. Al-Ghouti, A.H. El-Sheikh, Simultaneous determination of pesticides at trace levels in water using multiwalled carbon nanotubes as solid-phase extractant and multivariate calibration, Journal of Hazardous Materials, 169 (2009) 128-135.

[9] L. Latrous El Atrache, M. Hachani, B.B. Kefi, Carbon nanotubes as solid-phase extraction sorbents for the extraction of carbamate insecticides from environmental waters, International Journal of Environmental Science and Technology, 13 (2016) 201-208.

[10] M. Dong, Y. Ma, E. Zhao, C. Qian, L. Han, S. Jiang, Using multiwalled carbon nanotubes as solid phase extraction adsorbents for determination of chloroacetanilide herbicides in water, Microchimica Acta, 165 (2009) 123-128.

[11] Q. Zhou, Y. Ding, J. Xiao, Sensitive determination of thiamethoxam, imidacloprid and acetamiprid in environmental water samples with solid-phase extraction packed with multiwalled carbon nanotubes prior to high-performance liquid chromatography, Analytical and Bioanalytical Chemistry, 385 (2006) 1520-1525.

[12] I. Reinholds, I. Pugajeva, D. Zacs, E. Lundanes, J. Rusko, I. Perkons, V. Bartkevics, Determination of acidic non-steroidal anti-inflammatory drugs in aquatic samples by liquid chromatography-triple quadrupole mass spectrometry combined with carbon nanotubes-based solid-phase extraction, Environmental Monitoring and Assessment, 189 (2017) 568.

[13] A. Speltini, M. Maiocchi, L. Cucca, D. Merli, A. Profumo, Solid-phase extraction of PFOA and PFOS from surface waters on functionalized multiwalled carbon nanotubes followed by UPLC–ESI-MS, Analytical and Bioanalytical Chemistry, 406 (2014) 3657-3665.

[14] M.A. Salam, R. Burk, Solid phase extraction of polyhalogenated pollutants from freshwater using chemically modified multi-walled carbon nanotubes and their determination by gas chromatography, Journal of Separation Science, 32 (2009) 1060-1068.

[15] Y.-q. Cai, Y.-e. Cai, S.-f. Mou, Y.-q. Lu, Multi-walled carbon nanotubes as a solid-phase extraction adsorbent for the determination of chlorophenols in environmental water samples, Journal of Chromatography A, 1081 (2005) 245-247.

[16] J. Ma, R. Xiao, J. Li, J. Yu, Y. Zhang, L. Chen, Determination of 16 polycyclic aromatic hydrocarbons in environmental water samples by solid-phase extraction using multi-walled carbon nanotubes as adsorbent coupled with gas chromatography–mass spectrometry, Journal of Chromatography A, 1217 (2010) 5462-5469.

[17] W.-D. Wang, Y.-M. Huang, W.-Q. Shu, J. Cao, Multiwalled carbon nanotubes as adsorbents of solid-phase extraction for determination of polycyclic aromatic hydrocarbons in environmental waters coupled with high-performance liquid chromatography, Journal of Chromatography A, 1173 (2007) 27-36.

[18] S. Dahane, M.D. Gil García, M.J. Martínez Bueno, A. Uclés Moreno, M. Martínez Galera, A. Derdour, Determination of drugs in river and wastewaters using solid-phase extraction by packed multi-walled carbon nanotubes and liquid chromatography–quadrupole-linear ion trap-mass spectrometry, Journal of Chromatography A, 1297 (2013) 17-28.

[19] Q. Zhou, W. Wang, J. Xiao, J. Wang, G. Liu, Q. Shi, G. Guo, Comparison of the Enrichment Efficiency of Multiwalled Carbon Nanotubes, C18 Silica, and Activated Carbon as the Adsorbents for the Solid Phase Extraction of Atrazine and Simazine in Water Samples, Microchimica Acta, 152 (2006) 215-224.

[20] M.C. Prete, D.M. Dos Santos, L. Effting, C.R.T. Tarley, Preparation of Molecularly Imprinted Poly(methacrylic acid) Grafted on Iniferter-Modified Multiwalled Carbon Nanotubes by Living-Radical Polymerization for 17β-Estradiol Extraction, Journal of Chemical & Engineering Data, 64 (2019) 1978-1990.

[21] B. Lalović, T. Đurkić, M. Vukčević, I. Janković-Častvan, A. Kalijadis, Z. Laušević, M. Laušević, Solid-phase extraction of multi-class pharmaceuticals from environmental water samples onto modified multi-walled carbon nanotubes followed by LC-MS/MS, Environmental Science and Pollution Research, 24 (2017) 20784-20793.

[22] M.O. Barbosa, R.S. Ribeiro, A.R.L. Ribeiro, M.F.R. Pereira, A.M.T. Silva, Solid-phase extraction cartridges with multi-walled carbon nanotubes and effect of the oxygen functionalities on the recovery efficiency of organic micropollutants, Scientific Reports, 10 (2020) 22304.

[23] Q. Liu, J. Shi, J. Sun, T. Wang, L. Zeng, G. Jiang, Graphene and Graphene Oxide Sheets Supported on Silica as Versatile and High-Performance Adsorbents for Solid-Phase Extraction, Angewandte Chemie - International Edition, 50 (2011) 5913-5917.

[24] Y.-B. Luo, G.-T. Zhu, X.-S. Li, B.-F. Yuan, Y.-Q. Feng, Facile fabrication of reduced graphene oxide-encapsulated silica: A sorbent for solid-phase extraction, Journal of Chromatography A, 1299 (2013) 10-17.

[25] K.J. Huang, J. Li, Y.M. Liu, L. Wang, Sensitive determination of polycyclic aromatic hydrocarbons in water samples by HPLC coupled with SPE based on graphene functionalized with triethoxysilane, Journal of Separation Science, 36 (2013) 789-795.

[26] K.J. Huang, Y.J. Liu, J. Li, T. Gan, Y.M. Liu, Ultra-trace determination of polycyclic aromatic hydrocarbons using solid-phase extraction coupled with HPLC based on graphene-functionalized silica gel composites, Analytical Methods, 6 (2014) 194-201.

[27] A. Amiri, M. Baghayeri, F. Karimabadi, F. Ghaemi, B. Maleki, Graphene oxide/polydimethylsiloxane-coated stainless steel mesh for use in solid-phase extraction cartridges and extraction of polycyclic aromatic hydrocarbons, Microchimica Acta, 187 (2020) 213.

[28] X. Liu, H. Zhang, Y. Ma, X. Wu, L. Meng, Y. Guo, G. Yu, Y. Liu, Graphene-coated silica as a highly efficient sorbent for residual organophosphorus pesticides in water, Journal of Materials Chemistry A, 1 (2013) 1875-1884.

[29] Y. Yu, L. Wu, Application of graphene for the analysis of pharmaceuticals and personal care products in wastewater, Analytical and Bioanalytical Chemistry, 405 (2013) 4913-4919.

[30] A. Speltini, M. Sturini, F. Maraschi, L. Consoli, A. Zeffiro, A. Profumo, Graphene-derivatized silica as an efficient solid-phase extraction sorbent for pre-concentration of fluoroquinolones from water followed by liquid-chromatography fluorescence detection, Journal of Chromatography A, 1379 (2015) 9-15.

[31] Q. Han, Q. Liang, X. Zhang, L. Yang, M. Ding, Graphene aerogel based monolith for effective solid-phase extraction of trace environmental pollutants from water samples, Journal of Chromatography A, 1447 (2016) 39-46.

[32] J.M. Jiménez-Soto, S. Cárdenas, M. Valcárcel, Evaluation of carbon nanocones/disks as sorbent material for solid-phase extraction, Journal of Chromatography A, 1216 (2009) 5626-5633.

[33] A. Serrano, M. Gallego, Fullerenes as sorbent materials for benzene, toluene, ethylbenzene, and xylene isomers preconcentration, Journal of Separation Science, 29 (2006) 33-40.

[34] M. Kamran, M. Dauda, C. Basheer, M.N. Siddiqui, H.K. Lee, Highly efficient porous sorbent derived from asphalt for the solid-phase extraction of polycyclic aromatic hydrocarbons, Journal of Chromatography A, 1631 (2020) 461559.
